# Anharmonic Aspects in Vibrational Circular Dichroism Spectra from 900 to 9000 cm^–1^ for Methyloxirane and Methylthiirane

**DOI:** 10.1021/acs.jpca.2c05332

**Published:** 2022-09-20

**Authors:** Marco Fusè, Giovanna Longhi, Giuseppe Mazzeo, Stefano Stranges, Francesca Leonelli, Giorgia Aquila, Enrico Bodo, Bruno Brunetti, Carlo Bicchi, Cecilia Cagliero, Julien Bloino, Sergio Abbate

**Affiliations:** † Dipartimento di Medicina Molecolare e Traslazionale, Università di Brescia, Viale Europa 11, 25123 Brescia, Italy; ‡ Dipartimento di Chimica e Tecnologia del Farmaco, Università“La Sapienza”, P.le A. Moro 5, 00185 Roma, Italy; ¶ Dipartimento di Chimica, Università“La Sapienza”, P.le A. Moro 5, 00185 Roma, Italy; § ISMN-CNR, Università La Sapienza, P.le A. Moro 5, 00185 Roma, Italy; ∥ Dipartimento di Scienza e Tecnologia del Farmaco, Università degli Studi di Torino, Via Pietro Giuria 9,00124 Torino, Italy; ⊥ Scuola Normale Superiore, Piazza dei Cavalieri, 56125, Pisa, Italy; # Istituto Nazionale di Ottica (INO), CNR, Research Unit of Brescia, c/o CSMT, VIA Branze 45, 25123 Brescia, Italy; @ IOM-CNR, Laboratorio TASC, Basovizza, 34149 Trieste, Italy

## Abstract

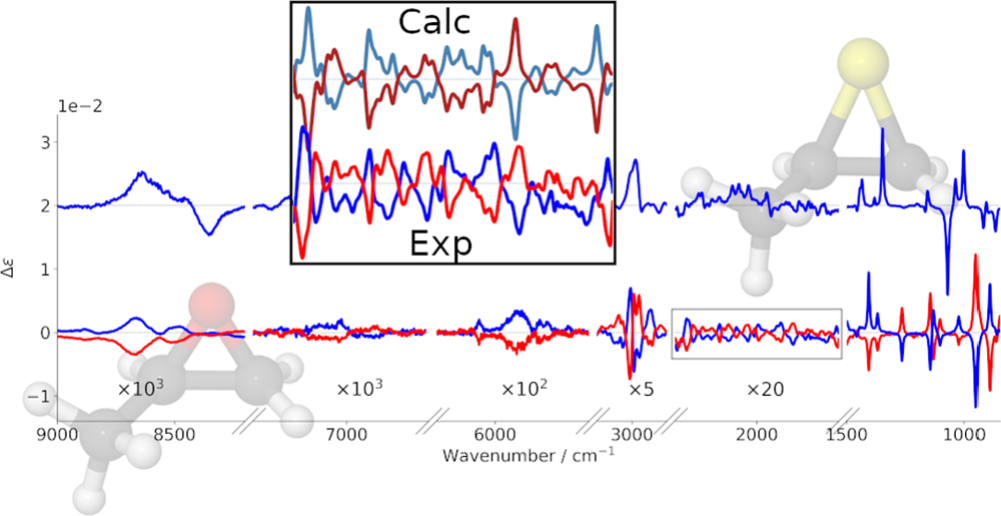

Vibrational circular dichroism (VCD) spectra and the corresponding IR spectra of the chiral isomers of methyloxirane and of methylthiirane have been reinvestigated, both experimentally and theoretically, with particular attention to accounting for anharmonic corrections, as calculated by the GVPT2 approach. De novo recorded VCD spectra in the near IR (NIR) range regarding CH-stretching overtone transitions, together with the corresponding NIR absorption spectra, were also considered and accounted for, both with the GVPT2 and with the local mode approaches. Comparison of the two methods has permitted us to better describe the nature of active “anharmonic” modes in the two molecules and the role of mechanical and electrical anharmonicity in determining the intensities of VCD and IR/NIR data. Finally, two nonstandard IR/NIR regions have been investigated: the first one about ≈2000 cm^–1^, involving mostly two-quanta bending mode transitions, the second one between 7000 and 7500 cm^–1^ involving three-quanta transitions containing CH-stretching overtones and HCC/HCH bending modes.

## Introduction

Anharmonicity is a very important issue to deal with for the accurate determination of several spectroscopically observable quantities, pertaining to rotational spectroscopy,^1,2^ to Raman and Raman optical activity (ROA), and to vibrational circular dichroism and infrared spectroscopies. In the last field, the seminal paper by Overend, Moscowitz, and associates^3^ first discussed the perturbative treatment at second order through the Van Vleck canonical S-transformation theory,^1^ to obtain dipole and rotational strengths corrected with mechanical anharmonic contributions; afterward the treatment has been extended to account also for electric anharmonicity on a single Morse oscillator^4^ and on two coupled oscillators.^5,6^ The problem is tackled in a more general way within the Generalized vibrational perturbation theory at the second order (GVPT2)^7−10^ implemented in the latest versions of Gaussian.^11^ The latter approach permits to account for both electrical and mechanical anharmonicities and also to treat resonances, be it of Fermi^12^ or Darling–Dennison^13^ types. Alternatively, the local-mode model is a more approximate but efficacious approach, suitable to describe the near-infrared (NIR) region, where only the large-amplitude and thus quite anharmonic XH-stretching modes are considered, both in the fundamental and overtone regions.^14−16^ The two methods were employed and the results were compared in a recent work from some of us dealing with the IR, VCD, NIR, and NIR-VCD spectra of chiral 2,3-butanediol and 1,2-*trans*-cyclohexanediol.^17^ Those two molecular systems have at least two accessible conformations, with the risk that the effect of anharmonicity is obscured by the conformational mobility, further complicated by the solvent dependence. For this reason, we have decided to treat conformationally rigid molecules, namely methyloxirane (propylene oxide) and methylthiirane (propylene sulfide), with the aim of better focusing onto the anharmonic effects. It is worth to mention that other cases for rigid molecules were partially treated with the anharmonic local mode approach, like camphor, (*S*)-dimethylallene and epichlorohydrin,^18−20^ and by VPT2 approach including methyloxirane in mid-IR region^21,22^ and camphor and α-pinene also in the first overtone region.^23^

Many vibrational chiroptical data have been presented through the years on the two molecules, especially for VCD^24−28^ but also for ROA,^29,30^ and some of those data will be considered here, together with our own data, either in the same regions or in the NIR region, for which no chiroptical data had ever been presented, to the best of our knowledge. We would also like to remind that the two molecules are important *per se*; indeed, methyloxirane has been discovered in the interstellar media as the first chiral molecule,^31^ by rotational spectroscopy. Referring to astrochemistry, we hope that the investigations presented here for the NIR region may provide a first suggestion to identify the presence of chiral molecules in the atmosphere of planets, much in the same way as Daniel et al.^32^ did to study the higher atmosphere of earth through local mode NIR absorption spectroscopy of OH/NH-containing species. Other forms of chiroptical methods have been employed on methyloxirane, like measuring and calculating the specific optical rotation (OR),^33−35^ also as determined by cavity ring-down polarimetry, allowing one to gain insight into structural and charge distribution aspects. Furthermore, in the ionization regime PECD (PhotoElectron Circular Dichroism) spectroscopy has been fruitfully applied to characterize enantiomers of methyloxirane free molecules.^36−39^ With the present study, a better characterization of the Atomic Polar Tensors (APT) and Atomic Axial Tensors (AAT) will be provided.^40^ The presentation of the results will proceed as follows. In the Experimental and Theoretical Computational Methods section, we will report comparatively the experimental VCD and IR spectra of the two compounds, pointing out similarities and differences in the two cases. In the same section we will briefly review the various computational approaches to deal with anharmonicity, which we had previously employed. In the Results and Discussion section the comparison of experimental and calculated VCD and IR/NIR spectra will be carried out, separating the IR and NIR regions, which notoriously are affected differently by anharmonic phenomena. The last section will contain the conclusions reached here on the two systems, with some attention to future perspectives, both in terms of methodology and of data.

## Experimental and Theoretical/Computational Methods

### Synthesis

In order to be able to carry out VCD measurements in the higher overtone NIR regions, we needed enantiomerically pure (*R*)-2-methylthiirane in substantial amount; thus, we reproduced its synthesis as previously reported in literature,^26,41^ obtaining (*R*)-2-methylthiirane from the conversion of (*S*)-2-methyloxirane. A full description of the synthetic procedure and the assessment of the optical purity is provided in section S1 of the Supporting Information. Optical rotatory dispersion (ORD) and electronic circular dichrosim (ECD) were also recorded (see Figures S4 and S5 in Supporting Information) and are in agreement with the data already reported in the literature.^42−44^ The two enantiomeric samples of methyloxirane were bought from Sigma-Aldrich and Alfa Aesar and used as received.

### Chiroptical Spectroscopic Methods

**Figure 1:**
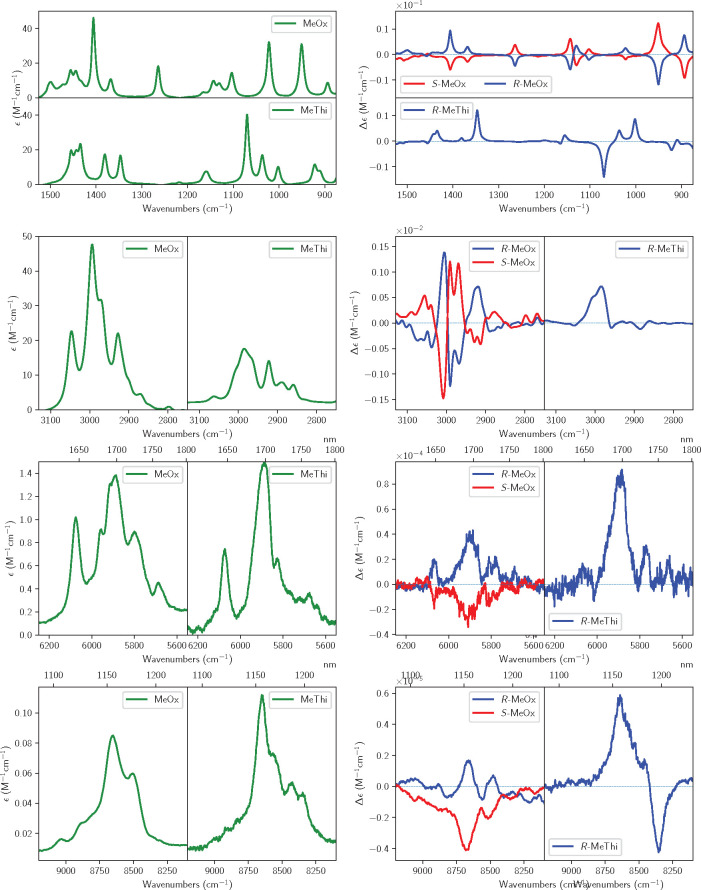
Experimental IR and VCD spectra of (*R*)-2-methylthiirane and 2-methyloxirane (enantiomers *R* and *S*). From the top, mid-IR (850–1500 cm^–1^), fundamental CH stretchings (2750–3050 cm^–1^), first overtone CH stretchings (5550–6250 cm^–1^), and second overtone CH stretchings (8100–9100 cm^–1^) spectroscopic regions are reported. The spectra have been recorded in 0.2 M/CCl_4_ solutions.

Several VCD measurements are present in the literature for methyloxirane and methylthiirane, both in the mid-IR and in the CH-stretching region.^24−28^ However, some data in the literature are for the neat liquid state and some other for solutions in organic solvents (e.g., CCl_4_, CS_2_, H_2_O). In order to be fully consistent, we decided to run our own spectra, always working with CCl_4_ solutions. For the mid-IR and CH-stretching regions, VCD measurements were carried out first with a JascoFVS4000 instrument and were repeated through the use of a JascoFVS6000 instrument. The two apparatuses are similar, i.e., they are FTIR instruments with added linear polarizer and ZnSe photoelastic modulator to achieve VCD measurements. The alignment and calibration procedure is different in the two cases, namely, it is more automatized and arranged through software in the second instrument. Final results were quite similar and we report data from the latest experiments. In both cases, a liquid N_2_-cooled HgCdTe (MCT) detector was employed for the mid-IR experiments (resolution 4 cm^–1^) and a liquid N_2_-cooled InSb detector for the CH-stretching fundamental region (resolution 8 cm^–1^). Solutions were contained in BaF_2_ 100 μm path length cells for the mid-IR and in quartz infrasil 1 mm path length cuvette for the CH-stretching region; unless otherwise specified, the concentration was 0.2 M/CCl_4_. 6000 scans were accumulated for each spectrum, and the reported data are averages over the accumulated spectra and the solvent spectra run in the same conditions were subtracted. Special care was paid to the region 1700–2300 cm^–1^, containing very weak nonfundamental transitions: 500 μm-BaF_2_ cells were employed, the rest of the conditions being the same. In the NIR region, we used a home-built dispersive instrument with a −20 °C cooled InGaAs detector, working in the range of 6250–12 500 cm^–1^, corresponding ca. to a 1600–800 nm interval and an extended InGaAs detector cooled at −30 °C in the range 5600–6250 cm^–1^, corresponding ca. to 1800–1600 nm.^20,45^ In the first CH-stretching overtone region (1800–1600 nm) 2 mm quartz cuvettes were employed, while for the second overtone (1300–1000 nm) region 2 cm quartz cuvettes were used. The concentration was 1.9 M and the number of accumulated scans was 5 with absorption baseline (ABL) spectra subtracted from CD spectra (see refs (45 and 20)) and solvent spectra run in the same condition were subtracted out in the final stage. For the first overtone region, presenting low signal-to-noise ratio, 48 scans were accumulated. The resolution was 4.5 nm in all the NIR range, corresponding to ≈15 cm^–1^ at 1700 nm and ≈30 cm^–1^ at 1200 nm.^45^ The most important and intense features of the IR and VCD spectra are reported for both enantiomers of methyloxirane and for the (*R*)-enantiomer of methylthiirane in Figure 1, from the mid-infrared (top) to the CH-stretching fundamental (second panel from top), to the first CH-stretching overtone (third panel from top) to the second CH-stretching overtone (bottom). Other regions were investigated and will be described in the following. Before interpreting VCD and IR spectra through DFT calculations, it may be worthwhile to just compare data for methyloxirane and methylthiirane, evidencing the major different features in the two cases. Mid-IR spectra: IR and VCD exhibit features with similar intensities, with the following differences. IR bands at ca. 1360 and 1050 cm^–1^ (showing positive and negative VCD respectively for (*R*)-methylthiirane) are typical of methylthiirane, while the 1400 cm^–1^ strong IR band (with positive VCD for (*R*)-methyloxirane) is typical of (*R*)-methyloxirane. Although only one atom differentiates the two molecules, their IR and VCD spectra are quite different. The largest observed *g*-ratio gets close to 1 × 10^–3^ for certain bands, which is rather large. The dissymmetry ratio *g* is defined as *g* = Δϵ/ϵ, and its value is expected to be of the order 10^–5^ in vibrational transitions.^46^ Fundamental CH-stretching spectra: here, observed *g*-ratios are more standard, limited above to 10^–4^. The major difference in the spectra of the two molecules is the fact that, overall, both IR and VCD in methylthiirane are weaker (by ca. one-half) than those for methyloxirane. The spectral band-shapes present also differences. The relative intensity of the highest frequency band is significantly larger in methyloxirane with respect to the most intense peak at ≈3000 cm^–1^. Furthermore, the IR spectrum of methylthiirane appears spread on a wider range of frequencies, suggesting participation from several close transitions. The picture is somehow reverted in the VCD spectra, where methyloxirane spectra are more structured. Nonetheless, some analogy between the systems may still be found. In particular, referring to the (*R*)-configuration and moving from high to low wavenumbers, a first weak negative band is followed by an intense positive signal in both systems.

First overtone CH-stretching spectra: the VCD spectrum is monosignate with three main positive features for (*R*)-methyloxirane, with *g*-values of the order of 0.2 × 10^–4^. Second overtone CH-stretching spectra: while the NIR spectra are similar in shape and overall intensity, with a central asymmetric doublet—though with additional features for (*R*)-methylthiirane at low energy—VCD features are quite different, with VCD for (*R*)-methylthiirane larger than for (*R*)-methyloxirane, the former consisting essentially in a negative–positive couplet (from low to high wavenumbers). In the case of (*R*)-methyloxirane, there appear two weak monosignate positive features corresponding to the main absorption features. The *g*-ratios are still 0.2 × 10^–4^ for (*R*)-methyloxirane, while they are larger for (*R*)-methylthiirane, reaching 0.8 × 10^–4^. All the data for the *g*-ratios are reported in section S2 of the Supporting Information.

### Theoretical Background and Computational Methods

#### The GVPT2 Approach: Basis and Advancements

Unless specified otherwise, calculations were performed with the Gaussian16 suite of quantum chemical programs.^11^ Based on the extensive benchmark analysis from ref (47), the combination of B3PW91^48^ functional with empirical dispersion (D3BJ) and the SNSD^8,47^ basis set was used, hereafter labeled PW91. Geometry optimizations were performed with tight convergence criteria (i.e., 1 × 10^–5^ hartree/bohr and 4 × 10^–5^ bohr on RMS forces and displacements, respectively, with thresholds for the maximum values being 1.5 times larger), and minima were confirmed by Hessian evaluations. Harmonic energies and intensities were obtained using analytic second derivatives of the energy and first derivatives of the properties of interest, whereas higher-order derivatives were computed through numerical differentiation using a step of 0.01  Å for the displacements along the mass-weighted normal coordinates. Harmonic frequencies, as well as energies and gradients, were also computed including solvent effects represented by the polarizable continuum model (PCM).^49,50^ Due to the finite differences procedure, the inclusion of solvent effects may lead to numerical instabilities in cubic and quartic force fields. Indeed, this effect was found to be particularly pronounced for XH stretchings since their motions are too fast to allow solvent equilibration.^23,50^ Since the harmonic spectra in gas-phase and in CCl_4_ shows negligible differences (see Figure S12 in Supporting Information), in order to retain consistency across simulations, we opted to employ gas-phase calculations in all the regions investigated. It should be noted that the problem of numerical stability, which may occur not only with the PCM cavity but also in the case of shallow PESs where the true minima may be difficult to reach, is relatively easy to assess. Indeed, the generation of all necessary anharmonic force constants and properties derivatives is partially redundant with nondiagonal quantities. For instance, a cubic force constant with respect to three different modes, *i*, *j*, and *k* is computed thrice, by displacement along the normal coordinates associated with each mode. It is thus possible to control the stability of the calculations by checking that the same value is constantly obtained for each alternative definition. In practice, because of the limits of numerical precision, some fluctuations may occur. A small value below 1 cm^–1^ for force constant has proven to be a good criteria of consistency and thus stability.

More accurate geometries were obtained by optimizing the molecular systems with the double hybrid revDSD-PBEP86 functional^51^ including empirical dispersion (D3BJ) in conjunction with the jun-cc-pVTZ^52,53^ basis set (rDSD in the following). Anharmonic calculations, up to three quanta transitions,^10^ were performed with the GVPT2 model as implemented in a development version of the Gaussian suite of programs.^54^ An issue that commonly plagues VTP2 calculations is the presence of resonances: in GVPT2, the diverging terms are removed from the perturbative expressions (IDVPT2^47^), and corrected in a second step through a reduced-dimensionality variational calculation (GVPT2). As described in ref (47), resonances are identified through two-step procedures, which account for the differences between energy and intensity. The employed parameters are reported in section S4 of Supporting Information. Calculation analysis and the plotting of the simulated spectra were supported by the ESTAMPES^55^ Python library for data processing and MATPLOTLIB graphical library.^56^

**Figure 2:**
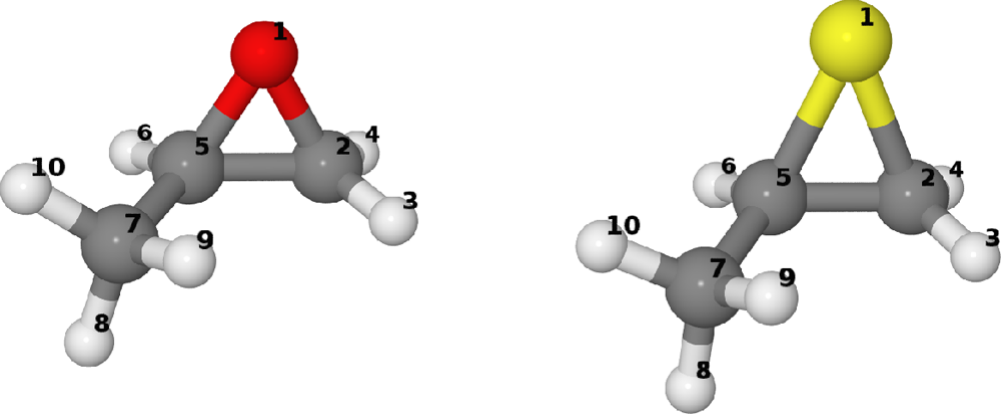
Chemical 3D structure and atom numbering of (*R*)-2-methylthiirane on the right and (*R*)-2-methyloxirane on the left.

In Figure 2, we provide the optimized geometries of (*R*)-2-methyloxirane and of (*R*)-2-methylthiirane. By simple inspection one may appreciate that the methyloxirane ring is almost an equilateral triangle, while the methylthiirane ring is a rather acute isosceles triangle. This may be further appreciated by looking at the data in Table 1. One may see that the CX (X = O, S) bond lengths are quite different for O and S, the latter being considerably longer (by ca. 0.4 Å), therefore showing a wider CX̂C in methyloxirane with respect to methylthiirane. However, and this will be interesting for the subsequent analysis, it is even more intriguing to notice that the lengths of the CH bonds directly connected to the epoxy (episulfide) ring, namely those for the CH_2_ and C*H groups, are shorter for the sulfide than for the oxide. Calculated CH bond lengths for the methyl group are more similar in the two cases and are more similar to the standard CH-bond lengths, e.g. in alkanes; in fact, they are slightly larger than 1.09 Å, while those for CH_2_ and C*H are closer to 1.08 Å. In correspondence of the shorter and tighter CH bonds, one may notice larger values for the harmonic vibration local-mode frequency ω_0_ (*vide infra*).

**Table 1:** Comparison of the Calculated Bond Lengths (Å) and Interbond Angle CXC (X = O, S) (deg) for Methyloxirane and Methylthiirane at the rDSD Level of Theory; Harmonic Wavenumbers (ω_0_) and Anharmonicity Constant (χ) as Defined in eq 2 and Obtained at PW91 Level Are also Given in Wavenumber Units (cm^–1^)^a^

	MeOx	MeThi	MeOx ω_0_	MeThi ω_0_	MeOx χ	MeThi χ
C_5_–C_2_	1.4635	1.4808				
C_7_–C_5_	1.5009	1.5063				
C_5_–X	1.4383	1.8318				
C_2_–X	1.4386	1.8272				
C_5_–H_6_	1.0872	1.0850	3115.01	3156.26	62.28	61.66
C_2_–H_4_	1.0848	1.0829	3145.23	3182.67	61.17	60.81
C_2_–H_3_	1.0855	1.0839	3139.60	3173.53	61.26	60.68
C_7_–H_8_	1.0928	1.0944	3089.49	3066.17	59.39	60.78
C_7_–H_9_	1.0911	1.0913	3107.14	3106.46	59.34	59.13
C_7_–H_10_	1.0920	1.0911	3091.87	3106.40	59.91	59.75
C_5_–X–C_2_	61.155	47.747				

a The atom numbering is reported in Figure 2.

In the following, vibrational transition current density (VTCD) vector fields were computed as reported in ref (57), the required electronic transition current density was computed for each state over a grid of points with a locally modified version of the Cubegen utility of Gaussian16 and saved as a discretized volumetric data set in plain-text cube files. 200 exited states were included in the sum-overstates procedure. 3D VTCD figures representing the vector field as streamtubes were obtained with the Mayavi package^58^ as reported in ref (57). Throughout the paper, the simulated spectra have been offset with respect to the experimental ones to improve the clarity of the presentation.

#### The Local Mode Model: Basis and Advancements

Here we briefly report the main equations we have employed to interpret IR and VCD spectra in the fundamental and overtone regions through the local-mode approximation. We use the notation previously employed by Paoloni et al.,^17^ but we remark that we go beyond the first order derivative in the Atomic Polar Tensor and Atomic Axial Tensor, in order to be able to account for the second overtone CH-stretching region, as previously done in refs (18 and 20), working on the basis of the results from Sage^59^ and Gallas.^60^ The transition energies from the ground to the excited states of one of the 6 CH stretching vibrations (*l* = 1, 6, related to the three groups CH_3_, CH_2_, and C*H) have the form:
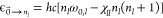
1with *n*_*l*_ the number of quanta associated with the CH bond *l*, and the parameters ω_0_ and χ (in wavenumber units) determined by the Morse potential parameters *D* and α,^61,62^ namely (omitting from now on the bond label *l*):
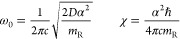
2*m*_R_ being the reduced mass of the CH bond, ca. (12/13) *u*, and the other constants besides *D* and α obvious fundamental constants. Following the procedure presented in refs (17 and 18), and reported in section S5 of Supporting Information, we obtained the values for ω_0_ and χ reported in Table 1 and commented above. Along the internal coordinate *z* corresponding to the stretching direction, the values of the *jz*-components (*j* = 1, 3) of the atomic polar tensor Π and their *z*-Taylor expansion and of the atomic axial tensor *A* and their *z*-Taylor expansion determining the electric and magnetic dipole transition moments, respectively, are required to compute the intensity of the properties and useful to define their role for the local mode calculations:
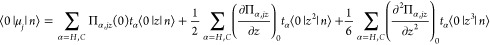
3
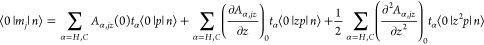
4where *p* is the conjugated moment to the *z*-coordinate along the CH-bond. The *t*_α_ are dimensionless displacements, being *t*_H_ ≈ (12/13) and *t*_C_ ≈ (−1/13).

In eqs 3 and 4, the Π_α,*jz*_(0) and *A*_α,*jz*_(0) are electrically harmonic terms, while the next two terms define *electrical anharmonicities*. In Table 2, we report the values for the transition moments appearing in eqs 3 and 4 formally evaluated at the harmonic level (harmonic oscillator eigenfunctions) and also including the contributions from the *mechanical anharmonicity*, that is, related to the eigenfunctions of the Morse oscillator. The latter contributions are given in terms of the (χ/ω_0_) parameter, which we were able to use for almost all transition moments. In all cases the moments were expressed in terms of the handy parameter *d*, which is a characteristic length, whose value is approximately 0.2 Å for CH stretching and is defined (in electrostatic cgs units) by
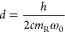
5where ω_0_ is the harmonic frequency in wavenumber units from eq 2. In Table 2, it is reassuring to notice that in all considered cases the values for the moments in the anharmonic case tend to those calculated for the harmonic case for (χ/ω_0_) → 0.

**Table 2:** Transition Integrals for Fundamental, First, and Second Overtones in the Local-Mode Representation^a^

quantity	harmonic case	anharmonic case
⟨0|*z*|1⟩	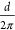	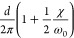
⟨0|*z*^2^|1⟩	0	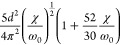
⟨0|*z*^3^|1⟩	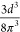	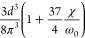
⟨0|*p*|1⟩	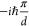	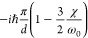
⟨0|*zp*|1⟩	0	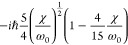
⟨0|*z*^2^*p*|1⟩	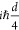	
⟨0|*z*|2⟩	0	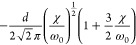
⟨0|*z*^2^|2⟩	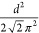	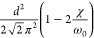
⟨0|*z*^3^|2⟩	0	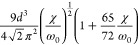
⟨0|*p*|2⟩	0	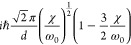
⟨0|*zp*|2⟩	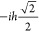	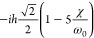
⟨0|*z*^2^*p*|2⟩	0	
⟨0|*z*|3⟩	0	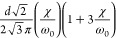
⟨0|*z*^2^|3⟩	0	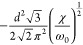
⟨0|*z*^3^|3⟩	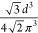	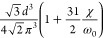
⟨0|*p*|3⟩	0	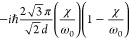
⟨0|*zp*|3⟩	0	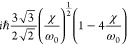
⟨0|*z*^2^*p*|3⟩	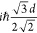	

a With respect to ref (17), the sign of χ is opposite and some expressions have been corrected.

## Results and Discussion

### Comparison of Calculated and Experimental Chiroptical Spectra

#### IR and VCD Spectra (900–1600 cm^–1^ Region)

**Figure 3:**
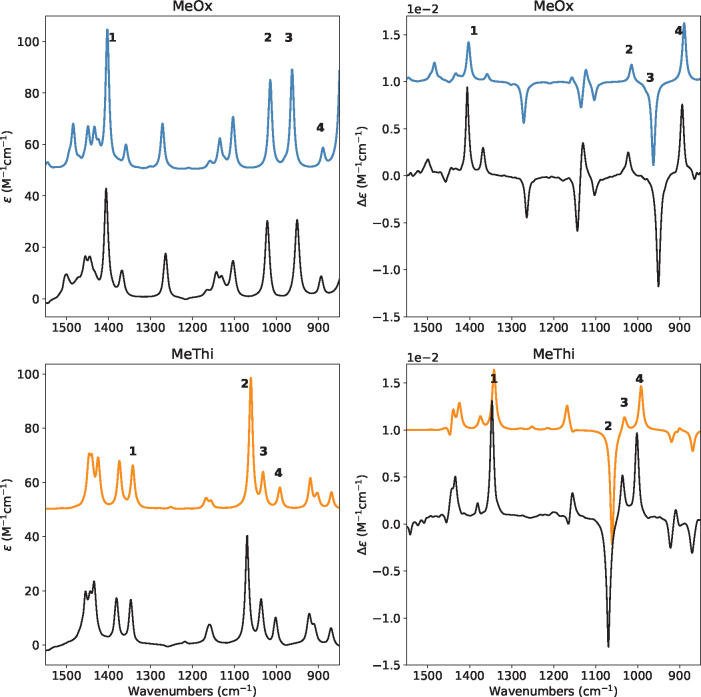
Comparison of the experimental IR and VCD spectra (black lines) of (*R*)-2-methylthiirane (bottom left and bottom right panels) and (*R*)-2-methyloxirane (top left and top right panels) with anharmonic calculations in the mid region (colored lines). Calculations were performed at the PW91 level. The spectra were simulated assigning Lorentzian distribution functions with 7 cm^–1^ of half-width at half-maximum. The reference VCD spectrum reported as experiment for the (*R*)-enantiomer of methyloxirane here is the semi-difference of the (*R*) and (*S*) VCD spectra of Figure 1.

In Figure 3, we report the results of the calculated mid-IR absorption and VCD spectra in the anharmonic case, and compare them to the corresponding experimental data. Results are in excellent agreement with the experimental spectra, in terms of sign (for VCD) and intensity (for both IR and VCD). Yet we notice that already the harmonic calculations for the same choice of functional/basis set performed excellently (Figure S10, in section S3). 0.98 scaling factor was applied to the latter computed spectra, while in the anharmonic case the results are plotted without any empirical correction. In passing, we also notice that the harmonic calculations by Polavarapu et al.^26^ and by Amos et al.,^63^ based on wave function theory, were very good as well. From this, we infer that anharmonicity, either mechanical or electrical, has little influence on the most prominent features of this region. However, the application of multiple scaling factors is required to have a good or at least acceptable correspondence between experimental and harmonically calculated spectra: in the present case the scaling factor was 0.98 for mid-IR and 0.96 for the fundamental CH-stretching region, respectively. Of course, the number of needed scaling factors may increase significantly with the size and number of conformers of the molecular systems. Anharmonic calculations, like the one presented here, allow one to avoid any arbitrariness related with employing empirical and variable scaling factors.^64^

An interesting feature of the VCD data of both molecules is that the *g*-ratio for most bands of this region is rather large, reaching the value 0.8 × 10^–3^ for the bands at ca. 1000 and 1360 cm^–1^ for (*R*)-2-methylthiirane (respectively marked as 4 and 1 in Figure 3) and at ca. 900 cm^–1^ for (*R*)-2-methyloxirane (marked as 4 in Figure 3). This remarkable behavior of the *g*-ratio had already been noticed by Polavarapu et al.^24,27^ Such value is indeed more typical of the electronic case than of the vibrational case. This is even more extraordinary considering the simplicity of the systems.

With the hope of learning something about the source of such strong VCD activity, we looked comparatively at the normal modes associated with four bands with high g and, in one case, with high VCD. Referring to Figure 3 we have band 1 at ≈1400 cm^–1^ for (*R*)-2-methyloxirane and at ≈1360 cm^–1^ for (*R*)-2-methylthiirane is HCH-bending (CH_2_) + C*H-bending + CH_3_-anstisymmetric bending + CC-stretching. Band 2 at ≈1020 cm^–1^ for (*R*)-2-methyloxirane is CH_2_–twisting + C*H-bending + CO-stretching. Band 2 at ≈1080 cm^–1^ for (*R*)-2-methylthiirane is CH_2_–rocking + C*H-bending + CS-stretching (strongest VCD for (*R*)-2-methylthiirane). Band 3 at ≈950 cm^–1^ for (*R*)-2-methyloxirane and at ≈1000 cm^–1^ for (*R*)-2-methylthiirane is CO-stretching + C*H-bending + CH_3_-umbrella (strongest VCD for (*R*)-2-methyloxirane) and CH_2_–wagging, respectively. Band 4 at ≈900 cm^–1^ for (*R*)-2-methyloxirane is CH_2_–rocking + C*H-bending + CO-stretching. Band 4 at ≈1000 cm^–1^ for (*R*)-2-methylthiirane is CH_2_–twisting + C*H-bending + CS-stretching. Bands 4 are the ones with the largest experimental *g*-ratio, about 0.8 × 10^–3^. Since the most intense bands seem to involve to some degree the CO/CS bond stretching (even though the normal modes which can be named as CO/CS stretching are found at lower frequencies,^24,27^ as may be checked from the harmonic normal modes), we thought that the involvement of the O/S atom in generating high *g*-ratio is due to the electrical rather than the mechanical anharmonicity.

**Figure 4:**
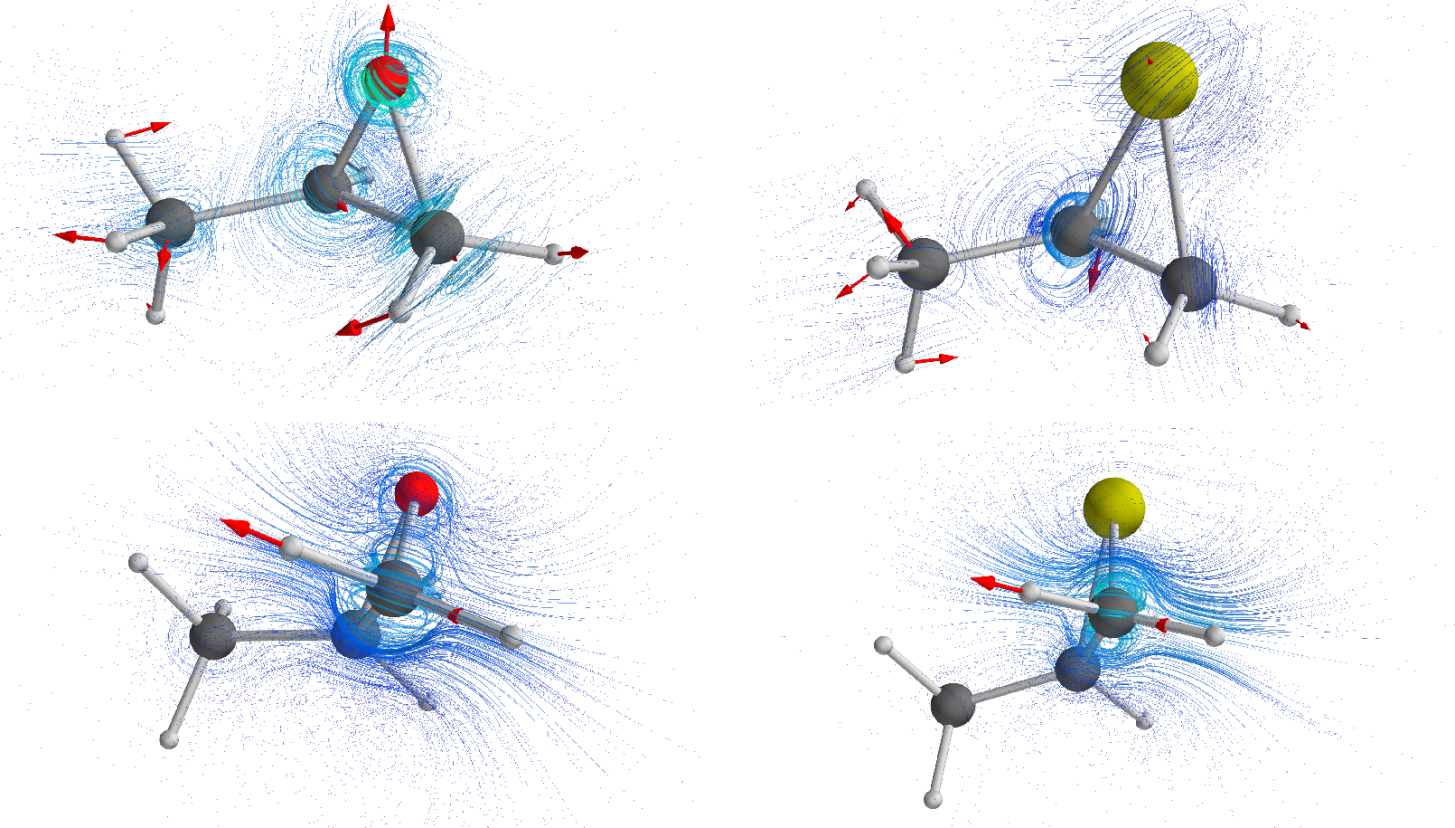
Top panels: 3D representations of the VTCD vector field for the high *g*-ratio normal modes of methyloxirane (6th normal mode, ≈900 cm^–1^, left) and methylthiirane (9th normal mode, ≈1010 cm^–1^, right). Bottom panels: 3D representations of the VTCD vector field for the CH_2_–antisymmetric stretching (24th normal mode) of methyloxirane (left) and methylthiirane (right). The red arrows depict the charge-weighed nuclear displacement vectors for the normal modes.

For this reason, we decided to plot in Figure 4 the current densities for the band 4 in the two cases, namely for band at 900 cm^–1^ for (*R*)-2-methyloxirane (6th normal mode) and 1000 cm^–1^ in (*R*)-2-methylthiirane (9th normal mode). We followed the method reported in ref (57), first presented by Nafie at al.^65,66^ To facilitate the comparison, we also plotted, in Figure 4, the current density for mode 24, which is found at the highest wavenumber. One may appreciate that the two mid-IR/high *g*-ratio transition exhibit the involvement of O and S atoms, even though curved patterns in the current density may be observed on all the heavy atoms of the rings. Circulation of charge is responsible for the electronic contribution to the magnetic dipole transition moment. In contrast, the antisymmetric stretching mode 24 presents high linear displacement current, associated with the electronic contribution to the electric dipole transition moment, from one hydrogen to the other in the CH_2_ unit, with smaller components on O/S and other heavy atoms; concomitantly its *g*-ratio is about 10^–4^.

#### IR and VCD Spectra (1700–2300 cm^–1^ Region): A Region for Overtones and Combinations Only

**Figure 5:**
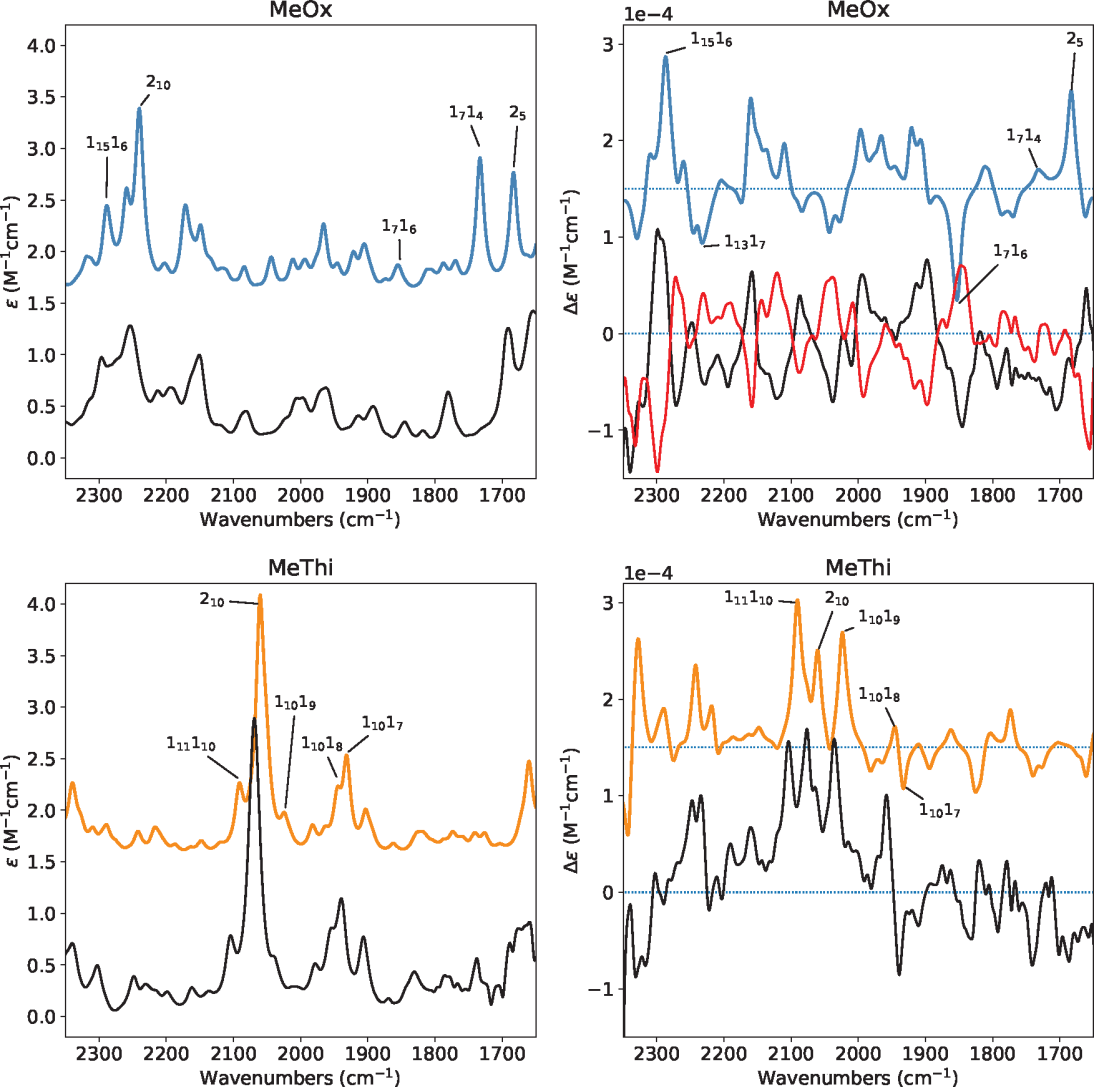
Comparison of the experimental IR and VCD spectra (black lines) of (*R*)-2-methylthiirane (bottom left and bottom right panels) and both enantiomers of 2-methyloxirane (top left and top right panels, *R*-enantiomer black line and *S* red line) with anharmonic calculations of *R*-enantiomers in the 2300–1600 region (colored lines). Calculations were performed at the PW91 level. The spectra were simulated assigning Lorentzian distribution functions with 7 cm^–1^ of half-width at half-maximum. The assignment of some transitions is also reported. “*n*_*m*_” represents the final vibrational state with *n* quanta associated with mode *m*.

In addition to the limitations discussed above, the harmonic approximation does not allow one to account for the contributions of the combinations and overtones to the spectral band-shape, contrary to anharmonic approaches like VPT2. In Figure 5, we compare the calculated and experimental IR absorption spectra in the 1600–2300 cm^–1^ range where only combinations and overtones are present. Stated otherwise, the region is void of fundamentals. At the GVPT2 level, we obtain an excellent agreement with the experiment. We wish to point out that the comparison of experimental and calculated data in this region had never been presented, to the best of our knowledge.

In this region, the most prominent transition is associated with the overtone band of the CH_2_ wagging—labeled as 2_10_ in Figure 5—which in methyloxirane is observed and calculated at an energy 180 cm^–1^ higher than in methylthiirane (i.e., 2070 and 2250 cm^–1^ respectively). Moreover, marked differences are present between the two systems. In methylthiirane the region is almost centered around the CH_2_ wagging overtones and the spectra are characterized by transitions involving the combination of the CH_2_ wagging mode with other CH bending modes close in energy. In contrast, in methyloxirane the role played by combinations involving the CH_2_ wagging is less pronounced and several combinations of CO stretching modes and ring deformation modes emerge. In Figure 5, some of the major transitions in both VCD and IR spectra are also reported and a description of the harmonic normal modes of the systems can be found in Table S1 in Supporting Information.

### NIR and NIR-VCD Spectra

#### CH Stretching Fundamentals and Overtones (2800–9000 cm^–1^ Regions)

**Figure 6:**
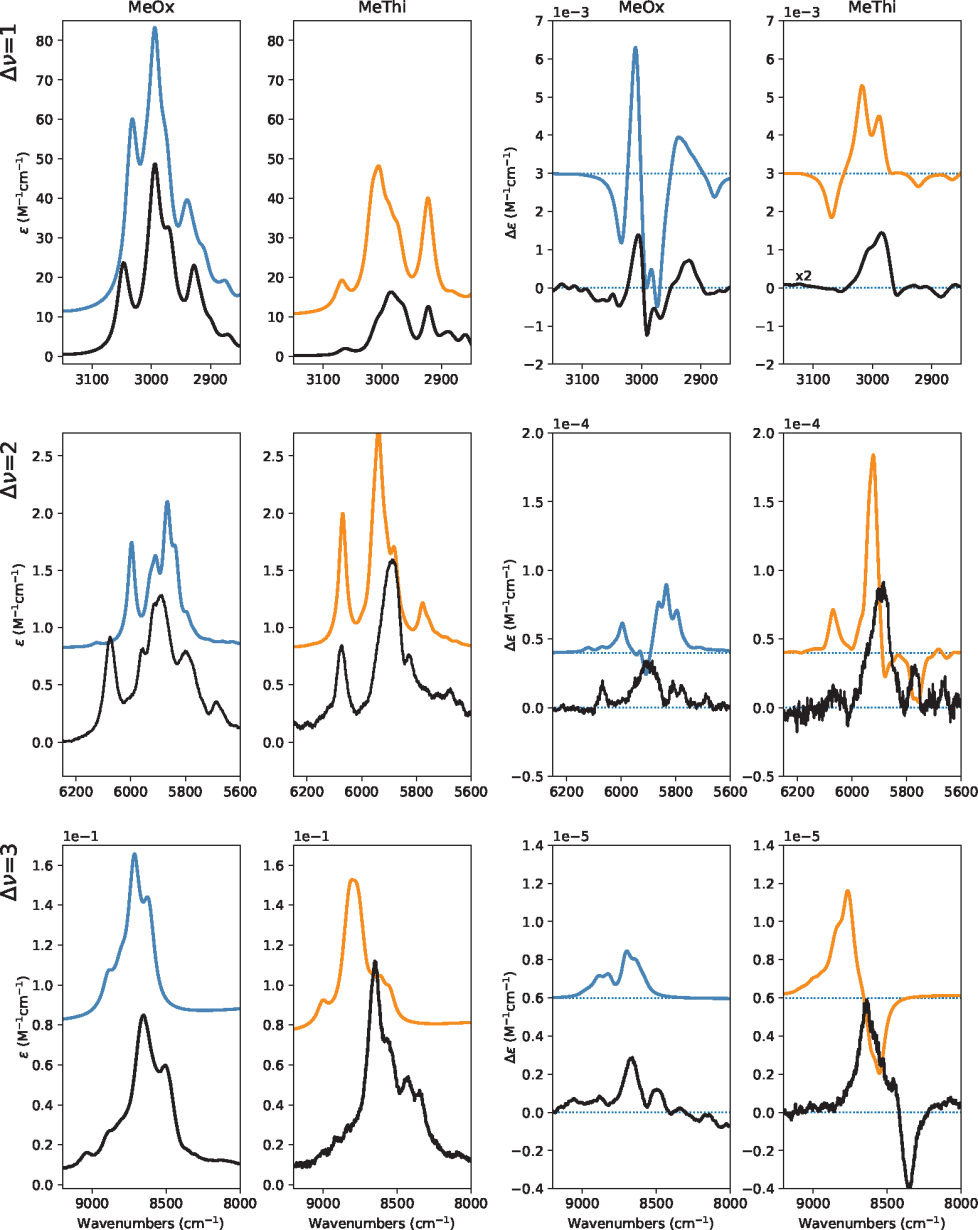
Comparison of experimental IR (first and second columns) and VCD (third and fourth columns) spectra (black lines) of (*R*)-2-methylthiirane and (*R*)-2-methyloxirane with anharmonic calculations of IR and VCD spectra in the CH regions (colored lines). Calculations were performed at the PW91 level. The spectra were simulated assigning Lorentzian distribution functions with 10, 15, and 40 cm^–1^ of half-width at half-maximum in the fundamental, first and second overtone regions, respectively. The reference VCD spectra reported as experiment for the (*R*)-enantiomer of methyloxirane here are the semi-difference of the (*R*) and (*S*) VCD spectra of Figure 1.

We next move to comment the results for the CH-stretching regions, comprised of the fundamental (Δν = 1) transitions, as well as of the first overtone (Δν = 2) and second overtone (Δν = 3) transitions. Unlike the mid-IR region, the *g*-ratios are of the order of 10^–4^, which is standard for the vibrational case. The same order of magnitude for the *g*-ratio is observed in the three regions, in accord with the conclusions drawn some time ago, based on perturbation theory, by Faulkner^3^ and by Abbate et al.^67^ The performance of the GVPT2 calculations is excellent, as may be seen from Figure 6

While the harmonic approximation performs well in the Δν = 1 region (see Figure S11), apart from an overestimation of the energy, which can be corrected with a scaling factor of 0.96, it is unable to reproduce the band-shape in the other two regions. By including the anharmonic effects, the sequences of signs in the VCD spectra are correctly reproduced, including the intensities, which are found in good agreement, even though slightly overestimated in the fundamental region with respect to the experiment, both in IR and VCD. A second minor issue is that the band positions for (*R*)-2-methylthiirane in the Δν = 1 and Δν = 3 (and presumably Δν = 2) are overestimated with respect to experiment. As for the assignment of the individual bands, in both molecules we have three main absorption features, a symmetrical narrow band at ca. 3050 cm^–1^, a broader central structure composed of multiple bands at 3000 cm^–1^ and a final feature at 2930 cm^–1^ followed by minor transitions. The first band, assigned to the CH_2_–antisymmetric stretching, ν_asym_(CH_2_), in both cases corresponds to a negative VCD signal. In the central portion, starting from higher energies, one encounters ν(C*H) and then another main broad band with two or more coalescing features, which are mainly associated with ν_asym_(CH_3_) and ν_sym_(CH_3_) modes with some contributions from ν(C*H). The corresponding VCD of this portion is composed of alternating sign features in (*R*)-2-methyloxirane with a slight prevalence of the positive sign and with a definite positive sign band in the VCD of (*R*)-2-methylthiirane. Finally, at the lowest frequency 2930 cm^–1^, one has a symmetric absorption band, followed by lower-intensity bands, which is associated with ν_sym_(CH_2_). The sign of the main VCD band in that region is positive for (*R*)-2-methyloxirane and negative for in (*R*)-2-methylthiirane.

Moving now to the first (Δν = 2) and second overtone (Δν = 3) CH-stretching regiona, we may see that, roughly speaking, the Δν = 2 absorption spectra of both (*R*)-2-methyloxirane and (*R*)-2-methylthiirane present one more band than the Δν = 3 absorption spectrum, namely, the higher frequency symmetrical feature at ca. 6100 cm^–1^. The rest of the spectra, though not identical, share similar patterns between the two regions. The overtones VCD spectra of (*R*)-2-methyloxirane and (*R*)-2-methylthiirane look different. The one of (*R*)-2-methyloxirane has positive bands for both Δν = 2 and Δν = 3, while in (*R*)-2-methylthiirane the calculated and observed VCD spectra have alternating signs at Δν = 3. The observed large noise in the methylthiirane Δν = 2 VCD spectrum allows only to unambiguously assign the most intense transition. In any case the higher frequency VCD positive feature observed and calculated for (*R*)-2-methyloxirane and calculated for (*R*)-2-methylthiirane corresponds to the isolated 6100 cm^–1^ absorption band. GVPT2 calculations perform excellently, reproducing most of the features observed in the spectra. Looking at the GVPT2 results for the band assignment of the calculated features in the 2- and 3-quanta transitions, we learn that the highest energy transitions (≈6100 cm^–1^) is the pure 2ν_asym_ of the CH_2_ stretching, whereas at lower frequencies, the weight of combination modes on the final spectrum is increasing. Also in the Δν = 3 region, the agreement with experiment, both in terms of energy and intensity, is remarkable. Though the simulation of NIR spectra in the three-quanta region within GVPT2 framework is gaining popularity in literature,^68−70^ it should be noted here that these are among the first GVPT2 VCD spectra reported. At Δν = 3, combinations and overtones of normal modes combine together to give the local-mode picture^16^ as first proposed by Lehmann^71^ and embraced by others^5^ (*vide infra*). The increasing contribution of combination bands moving from Δν = 2 to Δν = 3 may also be appreciated looking at Figure S13 in Supporting Information where line spectra are reported with different colors depending on the transitions.

In conclusion, we may say that the present calculations perform very well. Additionally, they help one to follow the normal mode-local mode transition (the normal mode regime may be still valid for Δν = 2, while the local mode one comes in at Δν = 3).

#### Stretching/Bendings Combinations (6000–8000 cm^–1^ Region)

**Figure 7:**
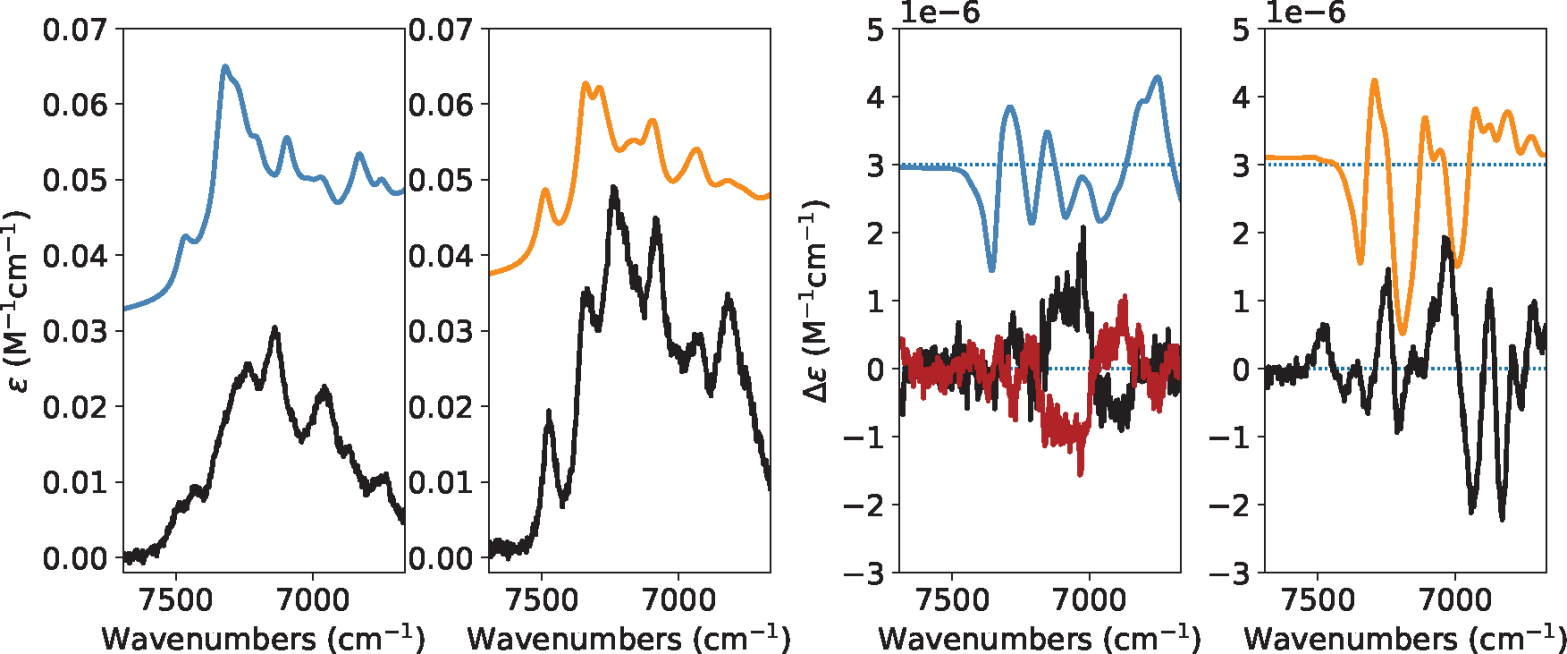
Comparison of the IR and VCD experimental and calculated anharmonic spectra of (*R*)-2-methyloxirane, (*R*)-2-methylthiirane (black line) and (*S*)-2-methylthiirane (red line) in the 6000–8000 cm^–1^ region. Calculations were performed at the PW91 level. The spectra were simulated assigning Lorentzian distribution functions with 30 cm^–1^ of half-width at half-maximum.

Before moving to the local-mode interpretation though, let us examine the intermediate spectroscopic region between Δν = 2 and Δν = 3, which, aided by simulations, is characterized by several combination transitions with the form of 2*i* + *k*, with *i* being of CH-stretching nature and *k* of bending nature. In Figure 7, we compare experimental and calculated NIR and NIR-VCD spectra in that region for both (*R*)-2-methyloxirane (first and third columns) and (*R*)-2-methylthiirane (second and fourth columns). Calculations, even based on empirical models, have been seldom presented for these bending-stretching combination region,^72^ but never, to the best of our knowledge, based on DFT or *ab initio* methods. In the present case, we have just verified that the highest frequency feature, at about 7469 cm^–1^ for (*R*)-2-methyloxirane and 7500 cm^–1^ for (*R*)-2-methylthiirane, are due to a combination involving two quanta from normal mode ν_asym_(CH_2_) and one quantum from the (HCH)-bending. The other features comprise other 3-quanta combinations, where C*H, CH_2_, and CH_3_-stretching modes combine variously. At 7325 cm^–1^ for (*R*)-2-methyloxirane and 7345 cm^–1^ for (*R*)-2-methylthiirane, we noticed combination transitions of the form *i* + *j* + *k*, with the three different normal modes being ν_asym_(CH_2_), ν_sym_(CH_2_), and a bending.

#### CH Stretching Fundamentals and Overtones (2800–8500 cm^–1^ Regions): The Local-Mode Interpretation

**Figure 8:**
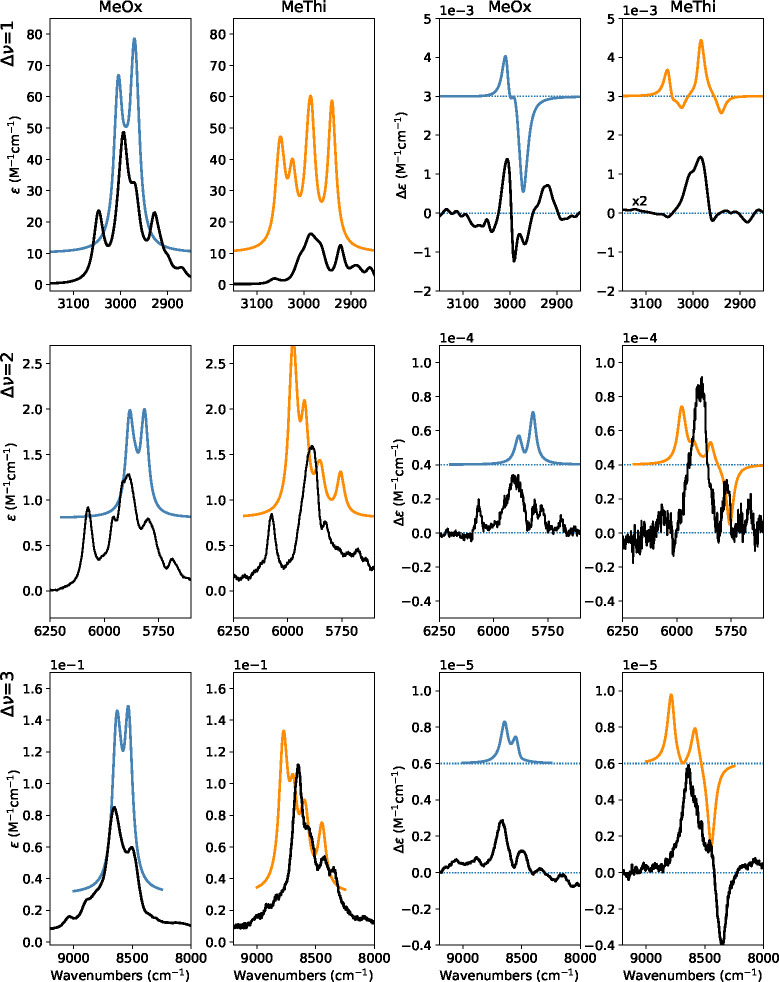
Comparison of the experimental IR (first and second columns) and VCD (third and fourth columns) spectra of (*R*)-2-methylthiirane (orange) and (*R*)-2-methyloxirane (blue) with the local-mode calculations in the CH-stretching regions. The spectra were simulated assigning Lorentzian distribution functions with 10, 15, and 40 cm^–1^ of half-width at half-maximum in the fundamental, first, and second overtone, respectively. The reference VCD spectra reported as experiment for the (*R*)-enantiomer of methyloxirane here are the semidifference of the (*R*) and (*S*) VCD spectra of Figure 1.

In Figure 8, we present the comparison of the experimental IR and VCD spectra for the fundamental (Δν = 1), the first (Δν = 2) and second overtone (Δν = 3) CH-stretching regions with the corresponding calculated ones. The energy of each transition associated with CH-stretching were obtained in the local-mode framework through the Birge–Sponer relation of eq 1 from the values of ω_0_ and χ reported in Table 1. The associated IR and VCD intensities were computed using eqs 3 and 4, where the required values of atomic polar tensors Π and atomic axial tensors *A* and their derivatives with respect to CH-bond elongations were obtained by polynomial fit of the values reported in Figures S14 and S15 for (*R*)-2-methyloxirane and (*R*)-2-methylthiirane, respectively. In Figure S16 of the Supporting Information, we present the calculated line spectra superimposed to the convoluted spectra, where the contributions of each CH stretching is highlighted with a color code.

The results are in fairly good agreement even with the local-mode approximation. Indeed, already in other molecules with multiple CH bonds, like camphor and camphorquinone^18,20^ or in OH cases,^17^ this had been proved, but this is the first instance for a systematic comparison with GVPT2 up to Δν = 3. The purely local-mode model can predict only overtone bands, disregarding combinations. For this reason, the number of transition calculated in each Δν region is always the same. As a result, two absorption bands are predicted for (*R*)-2-methyloxirane, the higher-frequency one collecting the CH_2_-local modes and the C*H isolated mode, the lower frequency one containing the CH_3_-local modes. For (*R*)-2-methylthiirane, four absorption bands are predicted, with the highest band still containing the CH_2_-local modes, while the three lower frequency ones correspond to the C*H and the CH_3_-local modes, the latter being “less degenerate” than for (*R*)-2-methyloxirane. Similar results are obtained for the VCD calculated spectra in the Δν = 1, Δν = 2, and Δν = 3 regions. Of course, the comparison with experimental data improves when increasing Δν, being optimal for Δν = 3. It is worth noting that all the anharmonic contributions, both mechanical and electrical, need to be accounted for to achieve a satisfactory agreement with the experimental spectra, as demonstrated in Figure S17, where we present the results obtained suppressing the contribution of electrical anharmonicity.

Comparing the two approaches (Figures 6 and 8), three aspects should be considered. First, intensities in the Δν = 1 region are overestimated in the local-mode model. This happened also in the GVPT2 approach, but to a lesser extent. Second, frequencies for (*R*)-2-methylthiirane are overestimated, and this imperfection is shared by the GVPT2 interpretation, most probably due to limitations in the chosen electronic structure calculation method. Finally, both in (*R*)-2-methyloxirane and (*R*)-2-methylthiirane, the observed highest frequency feature for Δν = 1 and Δν = 2 regions is not predicted by the local-mode approach, since it is due to the antisymmetric CH_2_ stretching fundamental (Δν = 1) and its overtone (Δν = 2), as noted above when commenting the GVPT2 results, which are satisfactory in this respect. Within the local-mode scheme, one should describe this feature at Δν = 2 as a combination with one quantum on each CH oscillator.

As a final comment, our work proves that the local mode regime takes place at Δν = 3, which is well represented either starting from the local mode basis or from normal mode basis as done in the previous section.

## Conclusions

In this work, we have interpreted the IR and VCD spectra of (*R*)-2-methyloxirane and (*R*)-2-methylthiirane from 900 to 9000 cm^–1^ with full account of anharmonic effects by means of the GVPT2 approach, through the use of Gaussian. We verified that in these cases the anharmonic perturbation over the mid-IR region (from 900 to 1600 cm^–1^) is of minor importance. In this region, the harmonic approximation already works pretty well, apart from a wavenumber scaling, with due account of the high values for the *g* dissymmetry ratio observed there (of the order of 10^–3^). To this instance, the visualization of the current densities allows one to appreciate that in that region a major role is played by either the oxygen or sulfur atom, even in absence of motions from these atoms. Conversely, due account of anharmonicity allows one to predict excellently the CH-stretching regions, from the fundamental (Δν = 1) to the next two overtone regions (Δν = 2 and Δν = 3). For the latter two regions and, to a limited extent, for the Δν = 1 region, the local-mode approximation gives also a fair account of the experimental data, despite disregarding any coupling between oscillators. Herein, we give the necessary transition integrals to calculate dipole and rotational strengths as simple functions of the parameter χ/ω_0_. This parameter has also been shown to be important to describe the transition from normal modes to local modes regime.^71,73^ The combined use of the GVPT2 and local mode methods permits one to appreciate how the normal modes couple among them, with modes more localized at higher energy, and vice versa. In other words and more specifically, we think that the comparison of the results from the two methods will allow in the future to build well-grounded harmonically coupled anharmonic-oscillator (HCAO) models,^14−16^ using as perturbation parameters (χ/ω_0_) or alternatively (λ/ω_0_) (λ being the frequency separation of normal modes, related to the oscillator coupling constants), as preliminarily done in refs (5 and 6). Finally other regions involving either combinations of pure deformation/bending modes (between 1700–2300 cm^–1^) or of combinations of CH-stretching overtone modes and bending modes (between 7000 and 7500 cm^–1^) have been quite satisfactorily interpreted, making us confident that the GVPT2 approach may now be proposed to practitioners as a ready-to-use tool, even at the analytical level. The results achieved on these two rigid molecules may pave the ground for the application of the method on more complex and flexible systems but also where the solvent may play a crucial role both in terms of direct interactions and of relative stability of the conformers.

## Supporting Information Available

Experimental procedures and characterization data (^1^H NMR, ^13^C NMR, GC) for (*R*)-2-methylthiirane; ORD and ECD spectra of (*R*)-2-methylthiirane; experimental *g*-ratio; calculated Harmonic spectra and normal mode assignment; stick spectra of GVPT2 calculation in the CH region; dependency of Π and *A* on the CH stretchings; stick spectra of CH region in local mode approach; and Cartesian coordinates of methylthiirane and methyloxirane (PDF)

## Dedication

We dedicate this work to professor Vincenzo Barone, friend to all of us, who is one of the pioneers of anharmonicity studies.

## References

[ref1] Nielsen H. H. The Vibration-Rotation Energies of Polyatomic Molecules Part II. Accidental Degeneracies. Phys. Rev. 1945, 68, 181–191. 10.1103/PhysRev.68.181.

[ref2] Puzzarini C.; Bloino J.; Tasinato N.; Barone V. Accuracy and Interpretability: The Devil and the Holy Grail. New Routes across Old Boundaries in Computational Spectroscopy. Chem. Rev. 2019, 119, 8131–8191. 10.1021/acs.chemrev.9b00007. 31187984

[ref3] Faulkner T. R.; Marcott C.; Moscowitz A.; Overend J. Anharmonic effects in vibrational circular dichroism. J. Am. Chem. Soc. 1977, 99, 8160–8168. 10.1021/ja00467a006.

[ref4] Polavarapu P. Vibrational optical activity of anharmonic oscillator. Mol. Phys. 1996, 89, 1503–1510. 10.1080/00268979609482553.

[ref5] Abbate S.; Gangemi R.; Longhi G. Dipole and rotational strengths for overtone transitions of a C2-symmetry HCCH molecular fragment using Van Vleck perturbation theory. J. Chem. Phys. 2002, 117, 7575–7586. 10.1063/1.1504705.

[ref6] Gangemi R.; Longhi G.; Abbate S. Calculated absorption and vibrational circular dichroism spectra of fundamental and overtone transitions for a chiral HCCH molecular fragment in the hypothesis of coupled dipoles. Chirality 2005, 17, 530–539. 10.1002/chir.20202. 16189835

[ref7] Barone V. Anharmonic vibrational properties by a fully automated second-order perturbative approach. J. Chem. Phys. 2005, 122, 014108 10.1063/1.1824881. 15638643

[ref8] Barone V.; Biczysko M.; Bloino J. Fully anharmonic IR and Raman spectra of medium-size molecular systems: accuracy and interpretation. Phys. Chem. Chem. Phys. 2014, 16, 1759–1787. 10.1039/C3CP53413H. 24346191 PMC4604664

[ref9] Barone V. The virtual multifrequency spectrometer: a new paradigm for spectroscopy. WIREs Comput. Mol. Sci. 2016, 6, 86–110. 10.1002/wcms.1238. PMC5654514 29075335

[ref10] Bloino J. A. VPT2 Route to Near-Infrared Spectroscopy: The Role of Mechanical and Electrical Anharmonicity. J. Phys. Chem. A 2015, 119, 5269–5287. 10.1021/jp509985u. 25535769

[ref11] Frisch M. J.; Trucks G. W.; Schlegel H. B.; Scuseria G. E.; Robb M. A.; Cheeseman J. R.; Scalmani G.; Barone V.; Petersson G. A.; Nakatsuji H. Gaussian16, rev. C.01; Gaussian, Inc.: Wallingford, CT, 2016.

[ref12] Fermi E. Über den Ramaneffekt des Kohlendioxyds. Z. Physik 1931, 71, 250–259. 10.1007/BF01341712.

[ref13] Darling B. T.; Dennison D. M. The Water Vapor Molecule. Phys. Rev. 1940, 57, 128–139. 10.1103/PhysRev.57.128.

[ref14] Child M. S.; Lawton R. T. Local and normal vibrational states: a harmonically coupled anharmonic-oscillator model. Faraday Discuss. Chem. Soc. 1981, 71, 273 10.1039/dc9817100273.

[ref15] Lawton R.; Child M. Local mode vibrations of water. Mol. Phys. 1979, 37, 1799–1807. 10.1080/00268977900101331.

[ref16] Henry B. R. Use of local modes in the description of highly vibrationally excited molecules. Acc. Chem. Res. 1977, 10, 207–213. 10.1021/ar50114a003.

[ref17] Paoloni L.; Mazzeo G.; Longhi G.; Abbate S.; Fusè M.; Bloino J.; Barone V. Toward Fully Unsupervised Anharmonic Computations Complementing Experiment for Robust and Reliable Assignment and Interpretation of IR and VCD Spectra from Mid-IR to NIR: The Case of 2,3-Butanediol and *trans* −1,2-Cyclohexanediol. J. Phys. Chem. A 2020, 124, 1011–1024. 10.1021/acs.jpca.9b11025. 31922423 PMC7993639

[ref18] Gangemi F.; Gangemi R.; Longhi G.; Abbate S. Calculations of overtone NIR and NIR-VCD spectra in the local mode approximation: Camphor and Camphorquinone. Vib. Spectrosc. 2009, 50, 257–267. 10.1016/j.vibspec.2009.01.004.

[ref19] Abbate S.; Longhi G.; Gangemi F.; Gangemi R.; Superchi S.; Caporusso A. M.; Ruzziconi R. Electrical and mechanical anharmonicities from NIR-VCD spectra of compounds exhibiting axial and planar chirality: The cases of (S)-2,3-pentadiene and methyl-d3 (R)- and (S)-[2.2]paracyclophane-4-carboxylate. Chirality 2011, 23, 841–849. 10.1002/chir.21013. 21898605

[ref20] Abbate S.; Castiglioni E.; Gangemi F.; Gangemi R.; Longhi G. NIR-VCD, vibrational circular dichroism in the near-infrared: Experiments, theory and calculations. Chirality 2009, 21, E242–E252. 10.1002/chir.20805. 19927373

[ref21] Bloino J.; Barone V. A second-order perturbation theory route to vibrational averages and transition properties of molecules: General formulation and application to infrared and vibrational circular dichroism spectroscopies. J. Chem. Phys. 2012, 136, 124108 10.1063/1.3695210. 22462836

[ref22] Kreienborg N. M.; Bloino J.; Osowski T.; Pollok C. H.; Merten C. The vibrational CD spectra of propylene oxide in liquid xenon: a proof-of-principle CryoVCD study that challenges theory. Phys. Chem. Chem. Phys. 2019, 21, 6582–6587. 10.1039/C9CP00537D. 30849167

[ref23] Cappelli C.; Bloino J.; Lipparini F.; Barone V. Toward Ab Initio Anharmonic Vibrational Circular Dichroism Spectra in the Condensed Phase. J. Phys. Chem. Lett. 2012, 3, 1766–1773. 10.1021/jz3006139. 26291857

[ref24] Pickard S. T.; Smith H. E.; Polavarapu P. L.; Black T. M.; Rauk A.; Yang D. Synthesis, experimental, and ab initio theoretical vibrational circular dichroism, and absolute configurations of substituted oxiranes. J. Am. Chem. Soc. 1992, 114, 6850–6857. 10.1021/ja00043a033.

[ref25] Dothe H.; Lowe M. A.; Alper J. S. Vibrational circular dichroism of methylthiirane. J. Phys. Chem. 1988, 92, 6246–6249. 10.1021/j100333a016.

[ref26] Polavarapu P. L.; Hess B. A.; Schaad L. J.; Henderson D. O.; Fontana L. P.; Smith H. E.; Nafie L. A.; Freedman T. B.; Zuk W. M. Vibrational spectra of methylthiirane. J. Chem. Phys. 1987, 86, 1140–1146. 10.1063/1.452257.

[ref27] Polavarapu P. L.; Pickard S. T.; Smith H. E.; Black T. M.; Rauk A.; Yang D. Vibrational circular dichroism and absolute configuration of substituted thiiranes. J. Am. Chem. Soc. 1991, 113, 9747–9756. 10.1021/ja00026a006.

[ref28] Losada M.; Nguyen P.; Xu Y. Solvation of Propylene Oxide in Water: Vibrational Circular Dichroism, Optical Rotation, and Computer Simulation Studies. J. Phys. Chem. A 2008, 112, 5621–5627. 10.1021/jp801996m. 18522383

[ref29] Ren X.; Lin W.; Fang Y.; Ma F.; Wang J. Raman optical activity (ROA) and surface-enhanced ROA (SE-ROA) of (+)-(R)-methyloxirane adsorbed on a Ag_20_ cluster. RSC Adv. 2017, 7, 34376–34381. 10.1039/C7RA04949H.

[ref30] Šebestík J.; Bouř P. Raman Optical Activity of Methyloxirane Gas and Liquid. J. Phys. Chem. Lett. 2011, 2, 498–502. 10.1021/jz200108v.

[ref31] McGuire B. A.; Carroll P. B.; Loomis R. A.; Finneran I. A.; Jewell P. R.; Remijan A. J.; Blake G. A. Discovery of the interstellar chiral molecule propylene oxide (CH_3_CHCH_2_O). Science 2016, 352, 1449–1452. 10.1126/science.aae0328. 27303055

[ref32] Daniel J. S.; Solomon S.; Kjaergaard H. G.; Schofield D. P. Atmospheric water vapor complexes and the continuum. Geophys. Res. Lett. 2004, 31, L06118 10.1029/2003GL018914.

[ref33] Kumata Y.; Furukawa J.; Fueno T. The Effect of Solvents on the Optical Rotation of Propylene Oxide. BCSJ. 1970, 43, 3920–3921. 10.1246/bcsj.43.3920.

[ref34] Müller T.; Wiberg K. B.; Vaccaro P. H. Cavity Ring-Down Polarimetry (CRDP): A New Scheme for Probing Circular Birefringence and Circular Dichroism in the Gas Phase. J. Phys. Chem. A 2000, 104, 5959–5968. 10.1021/jp000705n.

[ref35] Wilson S. M.; Wiberg K. B.; Cheeseman J. R.; Frisch M. J.; Vaccaro P. H. Nonresonant Optical Activity of Isolated Organic Molecules. J. Phys. Chem. A 2005, 109, 11752–11764. 10.1021/jp054283z. 16366625

[ref36] Turchini S.; Zema N.; Contini G.; Alberti G.; Alagia M.; Stranges S.; Fronzoni G.; Stener M.; Decleva P.; Prosperi T. Circular dichroism in photoelectron spectroscopy of free chiral molecules: Experiment and theory on methyl-oxirane. Phys. Rev. A 2004, 70, 014502 10.1103/PhysRevA.70.014502.

[ref37] Stranges S.; Turchini S.; Alagia M.; Alberti G.; Contini G.; Decleva P.; Fronzoni G.; Stener M.; Zema N.; Prosperi T. Valence photoionization dynamics in circular dichroism of chiral free molecules: The methyl-oxirane. J. Chem. Phys. 2005, 122, 244303 10.1063/1.1940632. 16035753

[ref38] Garcia G. A.; Nahon L.; Daly S.; Powis I. Vibrationally induced inversion of photoelectron forward-backward asymmetry in chiral molecule photoionization by circularly polarized light. Nat. Commun. 2013, 4, 2132 10.1038/ncomms3132. 23828557 PMC3715848

[ref39] Alberti G.; Turchini S.; Contini G.; Zema N.; Prosperi T.; Stranges S.; Feyer V.; Bolognesi P.; Avaldi L. Dichroism in core-excited and core-ionized methyloxirane. Phys. Scr. 2008, 78, 058120 10.1088/0031-8949/78/05/058120.

[ref40] Stephens P. J. Theory of vibrational circular dichroism. J. Phys. Chem. 1985, 89, 748–752. 10.1021/j100251a006.

[ref41] Kleiner C. M.; Horst L.; Würtele C.; Wende R.; Schreiner P. R. Isolation of the key intermediates in the catalyst-free conversion of oxiranes to thiiranes in water at ambient temperature. Org. Biomol. Chem. 2009, 7, 1397–1403. 10.1039/b820232j. 19300825

[ref42] Bendazzoli G.; Gottarelli G.; Palmieri P.; Torre G. The optical activity of R-(+)-propylen sulphide. Mol. Phys. 1973, 25, 473–477. 10.1080/00268977300100421.

[ref43] Breest A.; Ochmann P.; Pulm F.; Gödderz K.; Carnell M.; Hormes J. Experimental circular dichroism and VUV spectra of substituted oxiranes and thiiranes. Mol. Phys. 1994, 82, 539–551. 10.1080/00268979400100404.

[ref44] Crawford T. D.; Tam M. C.; Abrams M. L. The problematic case of (S)-methylthiirane: electronic circular dichroism spectra and optical rotatory dispersion. Mol. Phys. 2007, 105, 2607–2617. 10.1080/00268970701598097.

[ref45] Castiglioni E.; Lebon F.; Longhi G.; Abbate S. Vibrational Circular Dichroism in the Near Infrared: Instrumental Developments and Applications. Enantiomer 2002, 7, 161–173. 10.1080/10242430212877. 12206495

[ref46] Nafie L. A. Vibrational Optical Activity: Principles and Applications; John Wiley & Sons: Chichester, UK, 2011.

[ref47] Yang Q.; Mendolicchio M.; Barone V.; Bloino J. Accuracy and Reliability in the Simulation of Vibrational Spectra: A Comprehensive Benchmark of Energies and Intensities Issuing From Generalized Vibrational Perturbation Theory to Second Order (GVPT2). Front. Astron. Space Sci. 2021, 8, 665232 10.3389/fspas.2021.665232.

[ref48] Becke A. D. Density-functional thermochemistry. III. The role of exact exchange. J. Chem. Phys. 1993, 98, 5648–5652. 10.1063/1.464913.

[ref49] Mennucci B. Polarizable continuum model. WIREs Comput. Mol. Sci. 2012, 2, 386–404. 10.1002/wcms.1086.

[ref50] Cappelli C.; Lipparini F.; Bloino J.; Barone V. Towards an accurate description of anharmonic infrared spectra in solution within the polarizable continuum model: Reaction field, cavity field and nonequilibrium effects. J. Chem. Phys. 2011, 135, 104505 10.1063/1.3630920. 21932908

[ref51] Santra G.; Sylvetsky N.; Martin J. M. L. Minimally Empirical Double-Hybrid Functionals Trained against the GMTKN55 Database: revDSD-PBEP86-D4, revDOD-PBE-D4, and DOD-SCAN-D4. J. Phys. Chem. A 2019, 123, 5129–5143. 10.1021/acs.jpca.9b03157. 31136709 PMC9479158

[ref52] Dunning T. H. Gaussian basis sets for use in correlated molecular calculations. I. The atoms boron through neon and hydrogen. J. Chem. Phys. 1989, 90, 1007–1023. 10.1063/1.456153.

[ref53] Papajak E.; Zheng J.; Xu X.; Leverentz H. R.; Truhlar D. G. Perspectives on Basis Sets Beautiful: Seasonal Plantings of Diffuse Basis Functions. J. Chem. Theory Comput. 2011, 7, 3027–3034. 10.1021/ct200106a. 26598144

[ref54] Frisch M. J.; Trucks G. W.; Schlegel H. B.; Scuseria G. E.; Robb M. A.; Cheeseman J. R.; Scalmani G.; Barone V.; Petersson G. A.; Nakatsuji H. Gaussian Development Version, rev. J.19; Gaussian, Inc.: Wallingford, CT, 2021.

[ref55] Bloino J. estampes: A prototypical program for spectral analysis. github, 2020. https://github.com/jbloino/estampes (accessed 2022-09-02).

[ref56] Hunter J. D. Matplotlib: A 2D graphics environment. Comput. Sci. Eng. 2007, 9, 90–95. 10.1109/MCSE.2007.55.

[ref57] Fusè M.; Egidi F.; Bloino J. Vibrational circular dichroism under the quantum magnifying glass: from the electronic flow to the spectroscopic observable. Phys. Chem. Chem. Phys. 2019, 21, 4224–4239. 10.1039/C8CP06514D. 30747175

[ref58] Ramachandran P.; Varoquaux G. Mayavi: 3D Visualization of Scientific Data. Comput. Sci. Eng. 2011, 13, 40–51. 10.1109/MCSE.2011.35.

[ref59] Sage M. L. Morse oscillator transition probabilities for molecular bond modes. Chem. Phys. 1978, 35, 375–380. 10.1016/S0301-0104(78)85253-7.

[ref60] Gallas J. A. C. Some matrix elements for Morse oscillators. Phys. Rev. A 1980, 21, 1829–1834. 10.1103/PhysRevA.21.1829.

[ref61] Morse P. M. Diatomic Molecules According to the Wave Mechanics. II. Vibrational Levels. Phys. Rev. 1929, 34, 57–64. 10.1103/PhysRev.34.57.

[ref62] Haar D. T. The Vibrational Levels of an Anharmonic Oscillator. Phys. Rev. 1946, 70, 222–223. 10.1103/PhysRev.70.222.

[ref63] Amos R. D.; Handy N. C.; Palmieri P. Vibrational properties of (*R*)-methylthiirane from Mo/ller–Plesset perturbation theory. J. Chem. Phys. 1990, 93, 5796–5804. 10.1063/1.459575.

[ref64] Fusè M.; Mazzeo G.; Longhi G.; Abbate S.; Masi M.; Evidente A.; Puzzarini C.; Barone V. Unbiased Determination of Absolute Configurations by vis-à-vis Comparison of Experimental and Simulated Spectra: The Challenging Case of Diplopyrone. J. Phys. Chem. B 2019, 123, 9230–9237. 10.1021/acs.jpcb.9b08375. 31580674

[ref65] Freedman T. B.; Shih M.-L.; Lee E.; Nafie L. A. Electron Transition Current Density in Molecules. 3. Ab Initio Calculations for Vibrational Transitions in Ethylene and Formaldehyde. J. Am. Chem. Soc. 1997, 119, 10620–10626. 10.1021/ja9701568.

[ref66] Freedman T.; Lee E.; Nafie L. Vibrational transition current density in (2S,3S)-oxirane-d2: visualizing electronic and nuclear contributions to IR absorption and vibrational circular dichroism intensities. J. Mol. Struct. 2000, 550–551, 123–134. 10.1016/S0022-2860(00)00517-2.

[ref67] Abbate S.; Longhi G.; Santina C. Theoretical and experimental studies for the interpretation of vibrational circular dichroism spectra in the CH-stretching overtone region. Chirality 2000, 12, 180–190. 10.1002/(SICI)1520-636X(2000)12:4<180::AID-CHIR4>3.0.CO;2-2. 10790188

[ref68] Beć K. B.; Grabska J.; Ozaki Y.; Czarnecki M. A.; Huck C. W. Simulated NIR spectra as sensitive markers of the structure and interactions in nucleobases. Sci. Rep. 2019, 9, 17398 10.1038/s41598-019-53827-6. 31758033 PMC6874539

[ref69] Beć K. B.; Huck C. W. Breakthrough Potential in Near-Infrared Spectroscopy: Spectra Simulation. A Review of Recent Developments. Front. Chem. 2019, 10.3389/fchem.2019.00048. PMC6396078 30854368

[ref70] Grabska J.; Beć K. B.; Ozaki Y.; Huck C. W. Anharmonic DFT Study of Near-Infrared Spectra of Caffeine: Vibrational Analysis of the Second Overtones and Ternary Combinations. Molecules 2021, 26, 5212 10.3390/molecules26175212. 34500645 PMC8433751

[ref71] Lehmann K. K. On the relation of Child and Lawton’s harmonically coupled anharmonic–oscillator model and Darling–Dennison coupling ^a^). J. Chem. Phys. 1983, 79, 1098–1098. 10.1063/1.445849.

[ref72] Ricard-Lespade L.; Longhi G.; Abbate S. The first overtone of CH stretchings in polymethylene chains: A conformationally dependent spectrum. Chem. Phys. 1990, 142, 245–259. 10.1016/0301-0104(90)89086-6.

[ref73] Mills I.; Robiette A. On the relationship of normal modes to local modes in molecular vibrations. Mol. Phys. 1985, 56, 743–765. 10.1080/00268978500102691.

