# Supplementary material for: Anharmonic Aspects in Vibrational Circular Dichroism Spectra from 900 to 9000 cm–1 for Methyloxirane and Methylthiirane
Source: J Phys Chem A. 2022 Sep 20;126(38):6719–33. doi: 10.1021/acs.jpca.2c05332 (PMC9527749; doi:10.1021/acs.jpca.2c05332)
Supplement: Supplementary file 1 — jp2c05332_si_001.pdf [file jp2c05332_si_001.pdf]

Supporting Information: Anharmonic Aspects in Vibrational Circular Dichroism Spectra from 900 to 9000 cm^{-1} for Methyloxirane and Methylthiirane

Marco Fusè,[†] Giovanna Longhi,[†,#] Giuseppe Mazzeo,[†] Stefano Stranges,[‡,@] Francesca Leonelli,[¶] Giorgia Aquila,[¶] Enrico Bodo,[¶] Bruno Brunetti,[§] Carlo Bicchi,[‖] Cecilia Cagliero,[‖] Julien Bloino,[*,⊥] and Sergio Abbate[*,†,#]

†*Dipartimento di Medicina Molecolare e Traslazionale, Università di Brescia, Viale Europa 11, 25123 Brescia, Italy*

‡*Dipartimento di Chimica e Tecnologia del Farmaco, Università "La Sapienza", P.le A. Moro 5, 00185 Roma, Italy*

¶*Dipartimento di Chimica, Università "La Sapienza", P.le A. Moro 5, 00185 Roma, Italy*

§*ISMN-CNR, Università La Sapienza, P.le A. Moro 5, 00185 Roma, Italy*

‖*Dipartimento di Scienza e Tecnologia del Farmaco, Università degli Studi di Torino, Via Pietro Giuria 9,00124 Torino, Italy*

⊥*Scuola Normale Superiore, Piazza dei Cavalieri, 56125, Pisa, Italy*

#*Istituto Nazionale di Ottica (INO), CNR, Research Unit of Brescia, c/o CSMT, VIA Branze 45, 25123 Brescia, Italy*

@*IOM-CNR, Laboratorio TASC, Basovizza, Trieste, Italy*

E-mail: julien.bloino@sns.it; sergio.abbate@unibs.it

S1 Experimental Section

The conversion of (*S*)-2methyloxirane to (*R*)-2-methylthiirane was performed in water according to a known procedure[S1,S2] with ammonium thiocyanate at room temperature (20 °C). This synthetic route was selected, among those reported in the literature, because it involves a S_N2 mechanism that prevents the racemization of the chiral center, according to the following scheme:

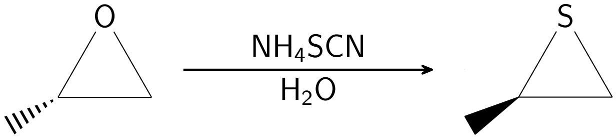

Scheme S1: Preparation of (*R*)-2-methylthiirane from (*S*)-2-methyloxirane in H_2O at 20 °C with ammonium thiocyanate.

The synthesized methylthiirane was purified with great difficulty. It degraded, indeed, very easily and was extremely volatile. Particularly, distillation of the reaction mixture by sample warming caused very low yields. Higher yields were only achieved by separation processes based on vacuum distillation in a low impedance transfer line between cold traps, whose temperature was controlled by thermostatic baths. The difference in vapor pressure, as a function of temperature, of solvents and the synthesized compound allowed a clean extraction of the methylthiirane. In this procedure monitoring of the nature of the extracted vapor in the transferred line, accomplished by recording He Iα (21.218 eV) photoelectron spectra (PES), was crucial.

S1.1 Synthesis of (*R*)-2-methylthiirane.

15 g of (S)-2-methyloxirane (0.26 mol), as purchased from Merck Life Science (99% purity), were introduced in a round-bottom flask with 500 mL distilled water and 115 g of ammonium thiocyanate (1.5 mol). The solution was magnetically stirred at 20 °C for four hours, then 50 mL of dodecane were added to quench the reaction. The solution was moved in a separating funnel to separate the organic phase, containing the synthesized compound, from the

aqueous solution. The 50 mL dodecane extraction from the aqueous solution was repeated twice. The organic phase, collecting the three extracted fractions, was dried over anhydrous Na_2SO_4. The solution thus obtained was filtrated and placed in a 250 mL two-necked flask that was connected to the vacuum transfer line. The most volatile species (atmospheric gases) were removed in the transfer line by two freezing-pumping cycles, where liquid nitrogen was used for freezing and the higher temperature was set at few degrees above the melting point of the solution (-5°C). Methylthiirane was extracted from the solution kept at -30 °C, and trapped in a 250 mL flask dipped in liquid nitrogen. The extraction, performed in static vacuum, required 45 hours. The pure (*R*)-2-methylthiirane was obtained with 29% yield. Chemical purity of the methylthiirane was checked by:

i ^{1}H-NMR (400.13 MHz, C_6D_6): δ 1.11 (3H, d, J= 5.74 Hz, CH_3), δ 1.65 (1H, dd of CH_2) and δ 2.02 (1H, dd of CH_2), δ 2.43 (1H, m, CH) (see Figure S1);

ii ^{13}C-NMR (100.61 MHz, C6D6): 30.0, 26.6, 21.8 (see Figure S2).

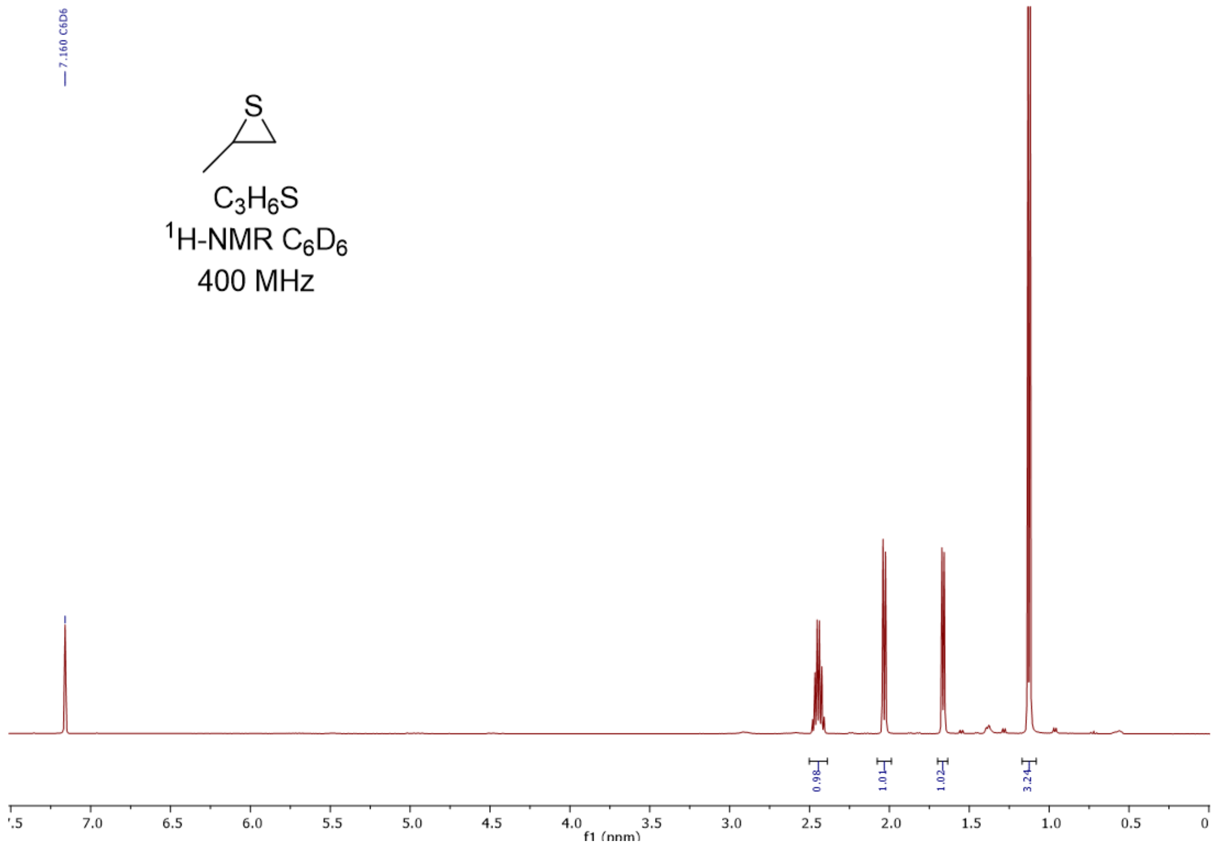

Figure S1: ^{1}H-NMR (C_6D_6, 400.13 MHz) spectrum of the synthesized (*R*)-2-methylthiirane.

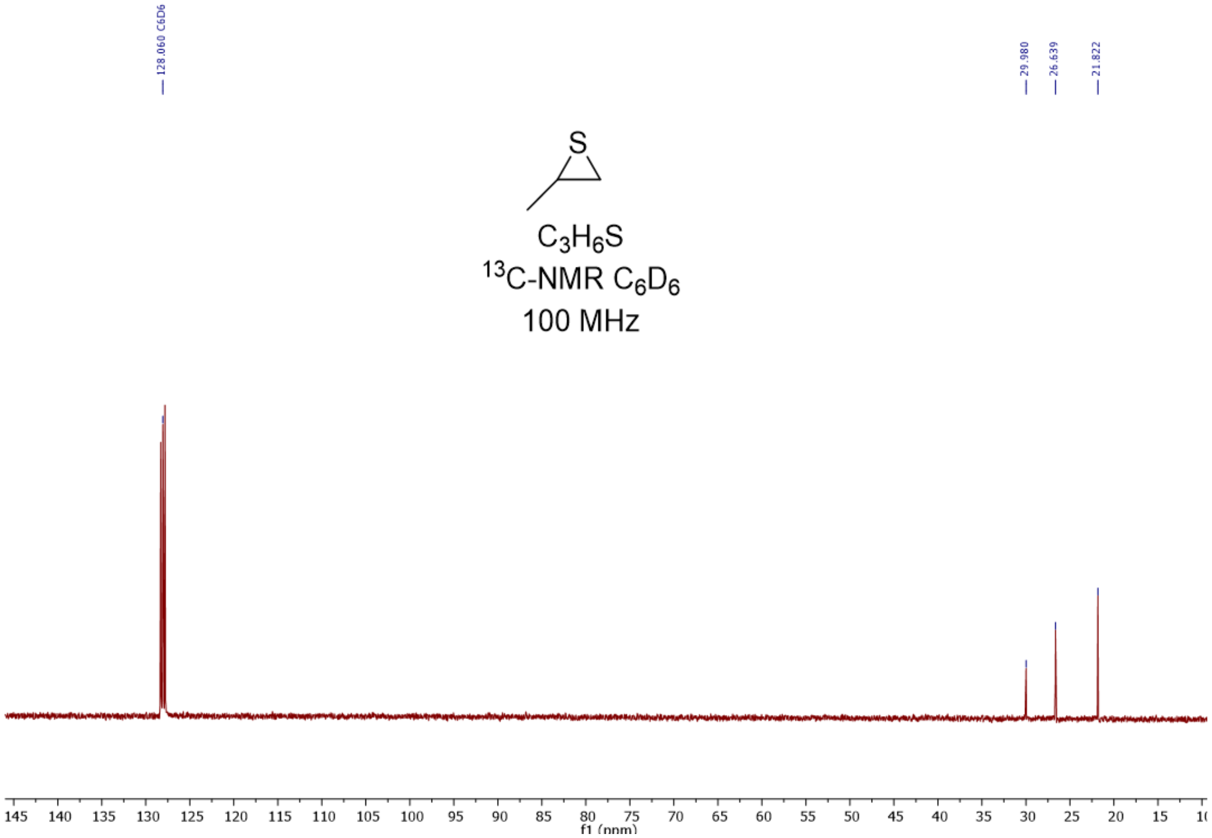

Figure S2: ^{13}C-NMR (C_6D_6, 100.61 MHz) spectrum of the synthesized (*R*)-2-methylthiirane.

S1.2 Enantioselective GC and GC-MS of methylthiirane

Since the enantiomeric purity affects the circular dichroism measurements, and because no data have been published on the enantiomeric purity of methylthiirane synthetized by this method, a direct analysis of the synthetized sample was performed by GC-MS (Gas Cromatography - Mass spectrometry) using a chiral stationary phase. An extensive study was needed to select the suitable stationary phase. The following cyclodextrin derivatives were tested as chiral selectors:

1. 2,3-di-*O*-ethyl-6-*O*-tert-butyldimethylsilil-β-cyclodextrin;
2. 2,3-di-*O*-methyl-6-*O*-tert-butyldimethylsilil-β-cyclodextrin,
3. 2,3-di-*O*-acetyl-6-*O*-tert-butyldimethylsilil-β-cyclodextrin,
4. 2,6-di-*O*-methyl-3-*O*-pentyl-β-cyclodextrin,
5. 2-*O*-ethyl-3-*O*-methyl-6-*O*-tert-butyldimethylsilil-β-cyclodextrin,
6. 2-*O*-methyl-3-*O*-ethyl-6-*O*-tert-butyldimethylsilil-β-cyclodextrin,
7. 2,3-di-*O*-acetyl-6-*O*-tert-heyxyldimethylsilil-γ-cyclodextrin.

The GC columns were coated with each chiral selector diluted at 30% in PS-086. The column providing the best results was that coated with 30% 2,3-di-O-acetyl-6-O-tert-butyldimethylsilil-β-cyclodextrin in PS086 (column 3). The results of the enantioselective analysis of the racemic methylthiirane (synthetized from racemic propylene oxide (Sigma-Aldrich, 99% purity) according to Scheme S1), and the synthesized *R*-enantiomer, are reported in Figure S3. A two-component fit of the GC chromatogram with asymmetric Gaussian functions gave the following results: i) for the racemic sample, 51.1% and 48.9% for the *S* and *R* enantiomers, respectively; for the chiral sample, 1.0% and 99.0%, for the *S* and *R* enantiomers, respectively. The purity of the synthetized methylthiirane was 98% e.e. Further technical details are given below.

GC and GC-MS systems: Analyses were carried out on a Shimadzu GC-FID 2010 unit equipped with Shimadzu GC Solution 2.53U software and a Shimadzu GC 2010 - Shimadzu QP2010-PLUS GC-MS system equipped with GCMS 2.51 software (Shimadzu, Milan, Italy).

Columns: the columns were coated with each chiral selector diluted at 30% in PS-086. Dimensions of columns coated with chiral selectors 1-6 were: length (l): 25m, inner diameter (d_c) 0.25mm, film thickness (d_f) 0.25 μm; column coated with chiral selector 7 was: l: 25m, 0.15mm d_c, 0.15μm d_f. The column providing the best results was that coated with 30% 2,3-di-*O*-acetyl-6-*O*-tert-butyldimethylsilil-β-cyclodextrin in PS086 (column 3).

Analysis conditions:

GC-FID conditions: temperatures: injector: 220°C, detector: 230°C, FID data acquisition rate: 50Hz. Injection mode: split; split ratio: 1:20, injection volume: 1 μL. All analyses were carried out with hydrogen as carrier gas helium in constant linear velocity mode; initial flow rate: 1 mL/min. Temperature program: from 50 to 220 °C at 2 °C/min.

GC-MS conditions temperatures: injector: 220°C, transfer line: 230 °C; ion source: 200 °C; carrier gas: He, flow control mode: constant linear velocity, initial flowrate: 1 mL/min. The MS operated in electron ionization mode (EI) at 70 eV, scan rate: 4.5 scan/sec, mass range: 35-350 m/z.

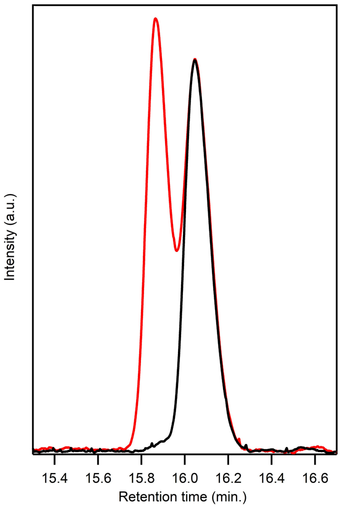

Figure S3: GC chromatograms of methythiirane samples: a) racemic sample (red curve), synthesized (*R*)-methylthiirane (black curve). Both chromatograms are obtained using column 3.

S1.3 Methylthiirane Optical Rotatory Dispersion

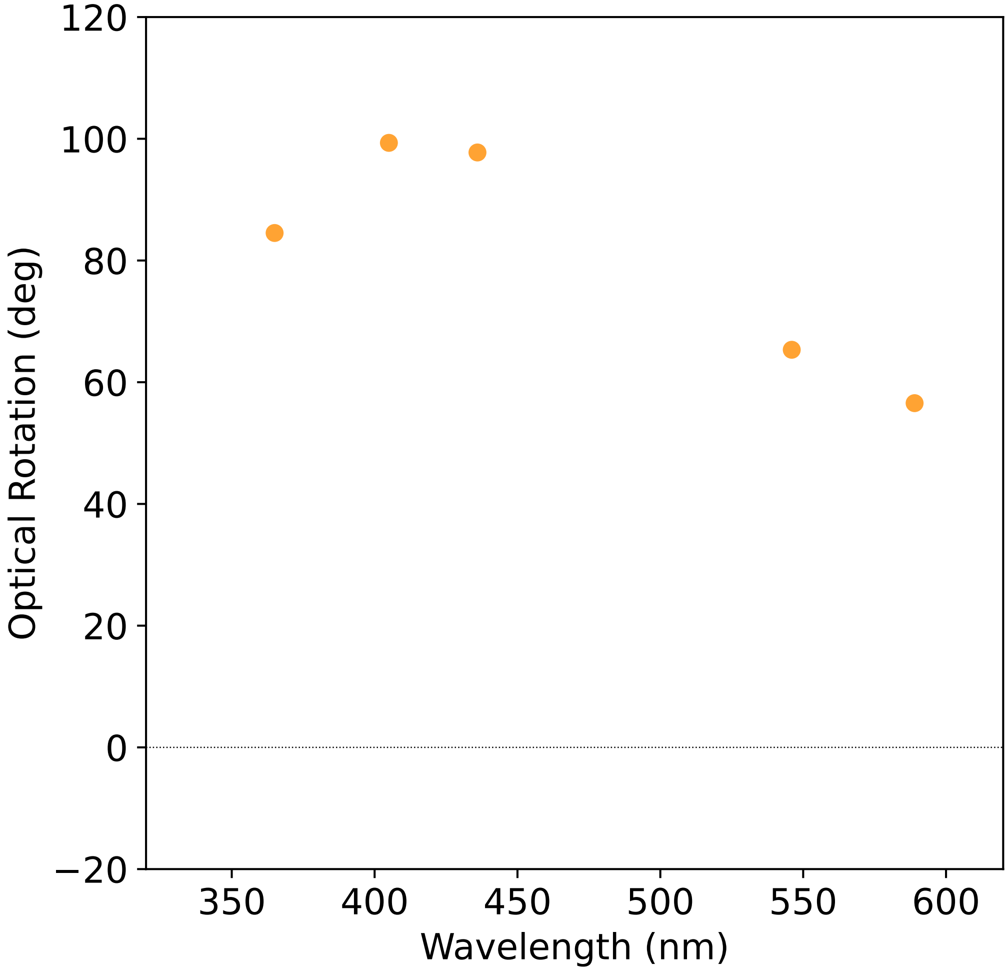

Figure S4: Optical rotatory dispersion of (*R*)-2-Methylthiirane in CCl_4 solution (c 24.4).

S1.4 Methylthiirane Electronic Circular Dichroism

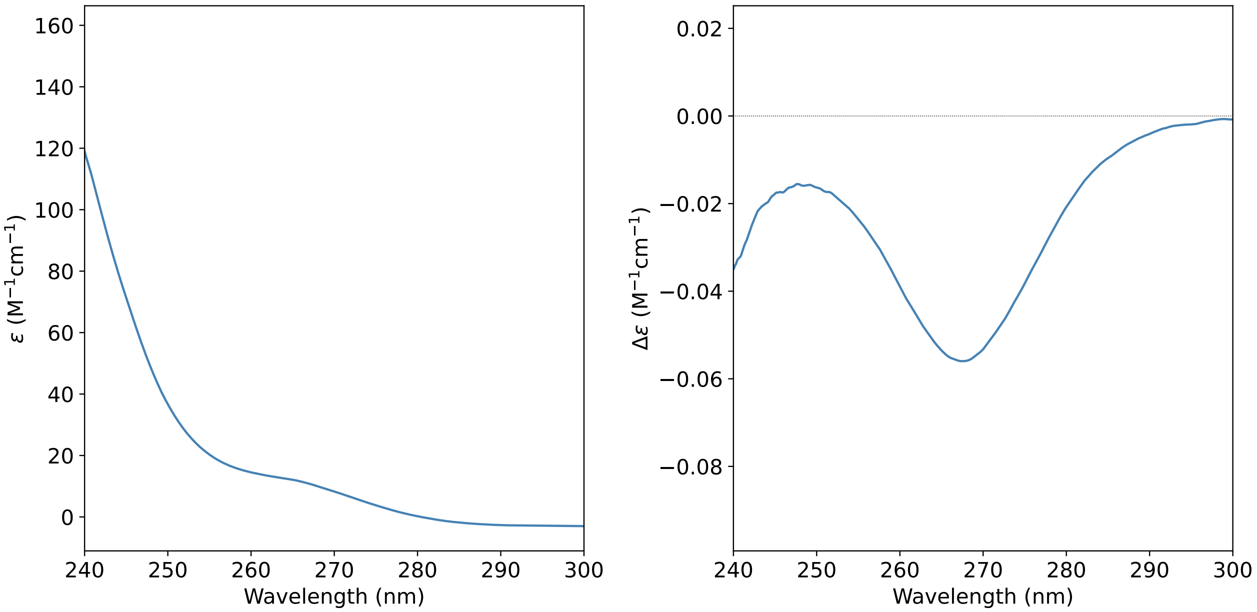

Figure S5: UV-Vis absorption and ECD of (*R*)-2-Methylthiirane in CCl_4 solution (C = 1.9M).

S2 Experimental g-ratios

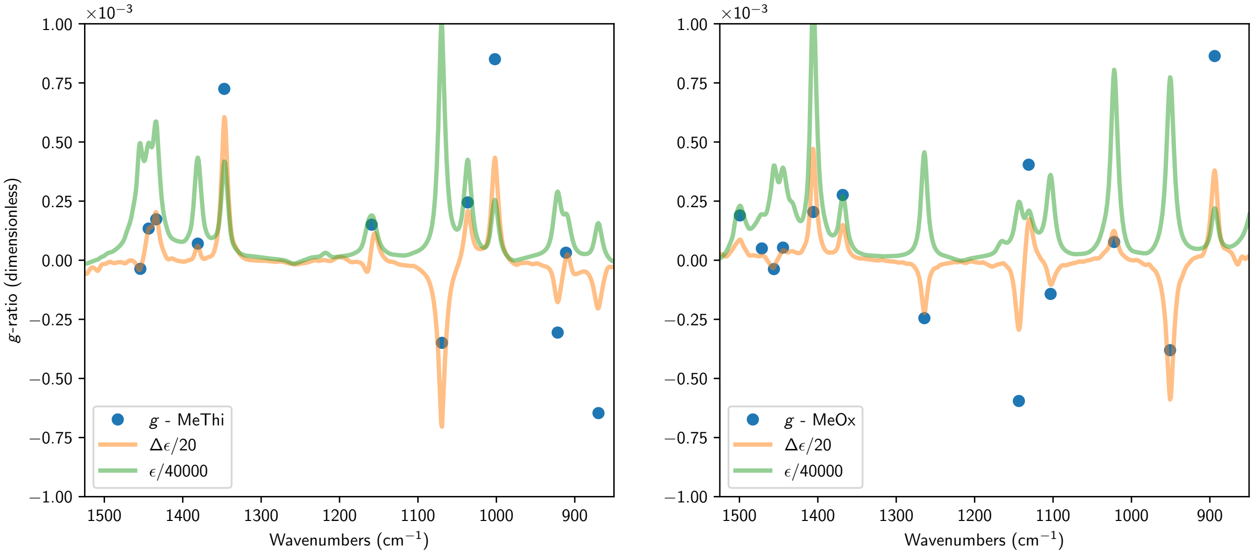

Figure S6: g-ratios of the observed transitions plotted as blue dots in the mid-IR region. The IR and VCD spectra are also reported, with appropriate scaling factors applied to fit the y-axis scale.

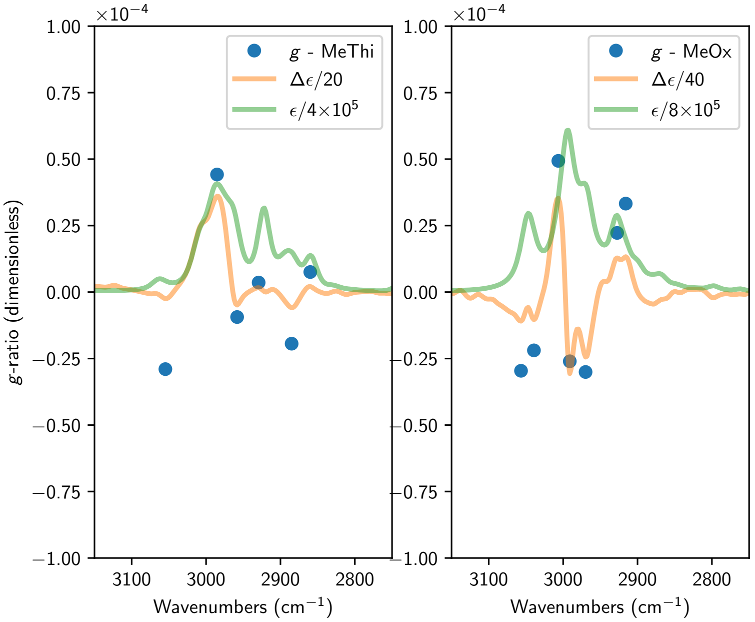

Figure S7: g-ratios of the observed transitions plotted as blue dots in the fundamental CH-stretching region. The IR and VCD spectra are also reported, with appropriate scaling factors applied to fit the y-axis scale.

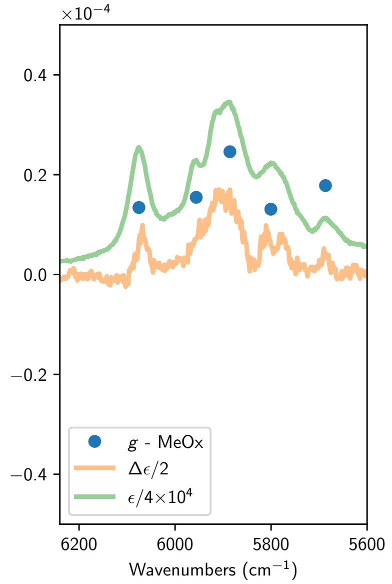

Figure S8: g-ratios of the observed transitions plotted as blue dots in the first overtone CH-stretching region. The IR and VCD spectra are also reported, with appropriate scaling factors applied to fit the y-axis scale.

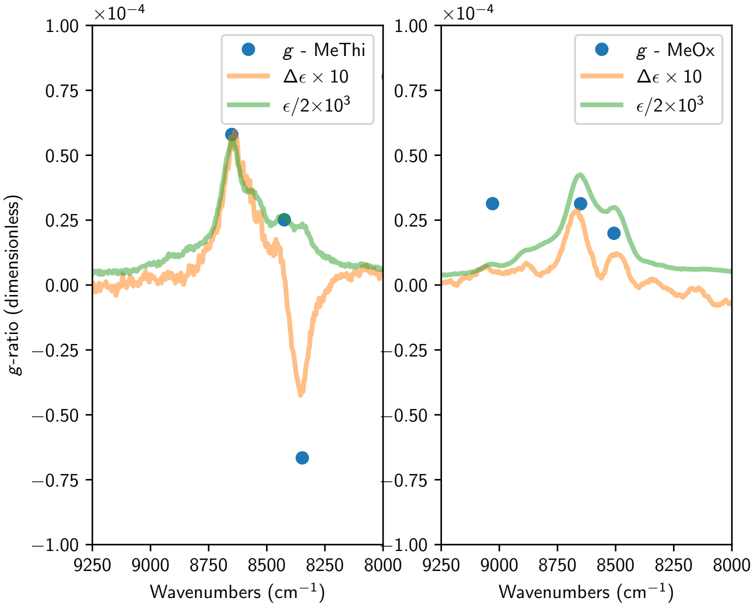

Figure S9: g-ratios of the observed transitions plotted as blue dots in the second overtone CH-stretching region. The IR and VCD spectra are also reported, with appropriate scaling factors applied to fit the y-axis scale.

S3 Harmonic Simulations

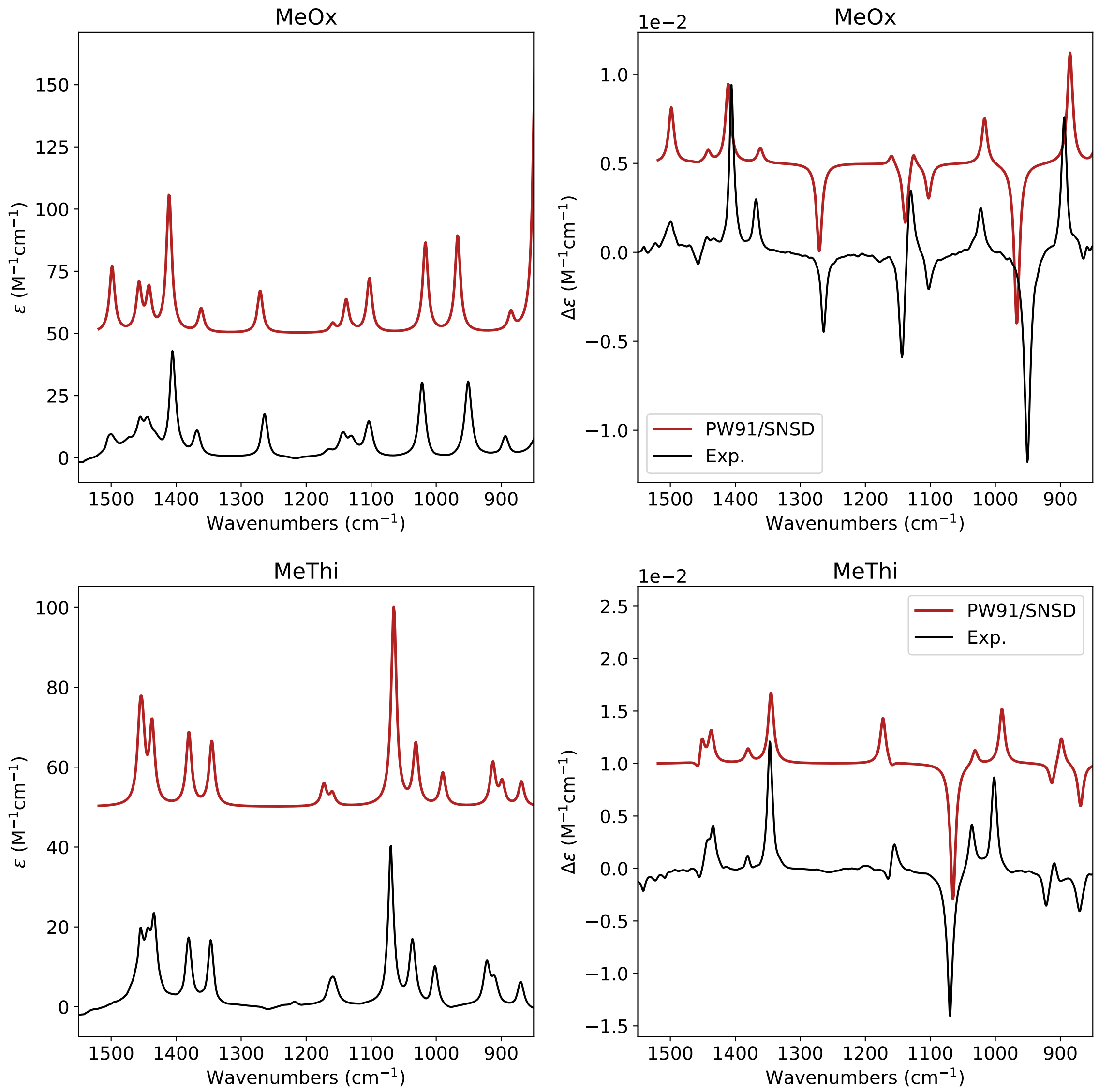

Figure S10: Comparison of the experimental (black lines) and harmonic IR and VCD spectra of (R)-2-methyloxirane (top panels) and (R)-2-methylthiirane (bottom panels) in the mid-IR region. Calculations were performed at the PW91 level of theory. The spectra were simulated by assigning Lorentzian distribution functions with 7 cm^{-1} of half-width at half-maximum. A scaling factor of 0.98 was applied.

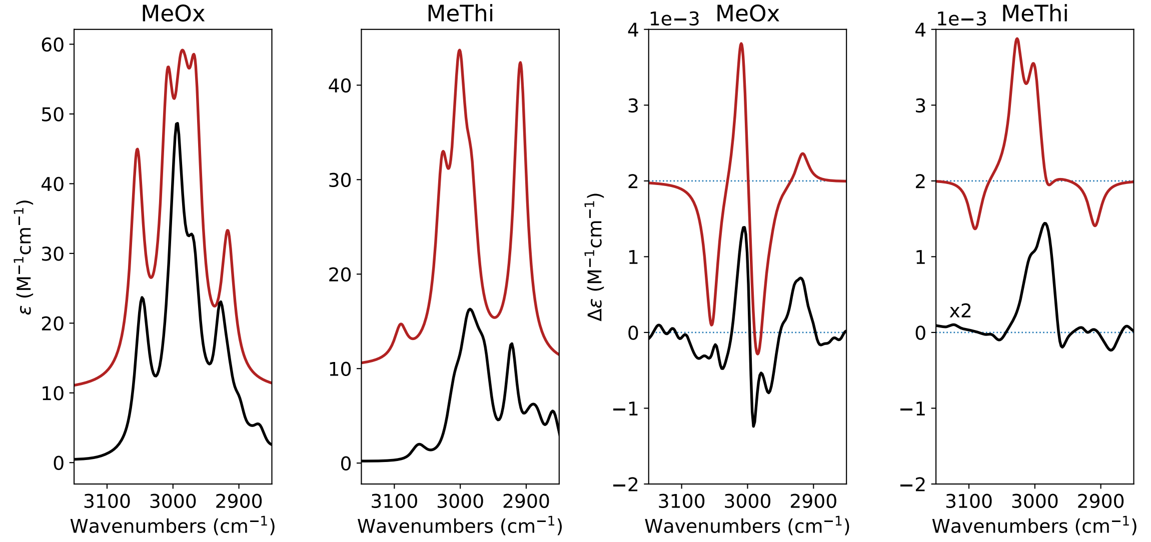

Figure S11: Comparison of the experimental and harmonic IR and VCD spectra (black lines) of (R)-2-methyloxirane (first and third column) and (R)-2-methylthiirane (second and fourth column) in the fundamental CH stretching region. Calculations were performed at the PW91 level of theory. The spectra were simulated by assigning Lorentzian distribution functions with 12 cm^{-1} of half-width at half-maximum. A scaling factor of 0.96 was applied.

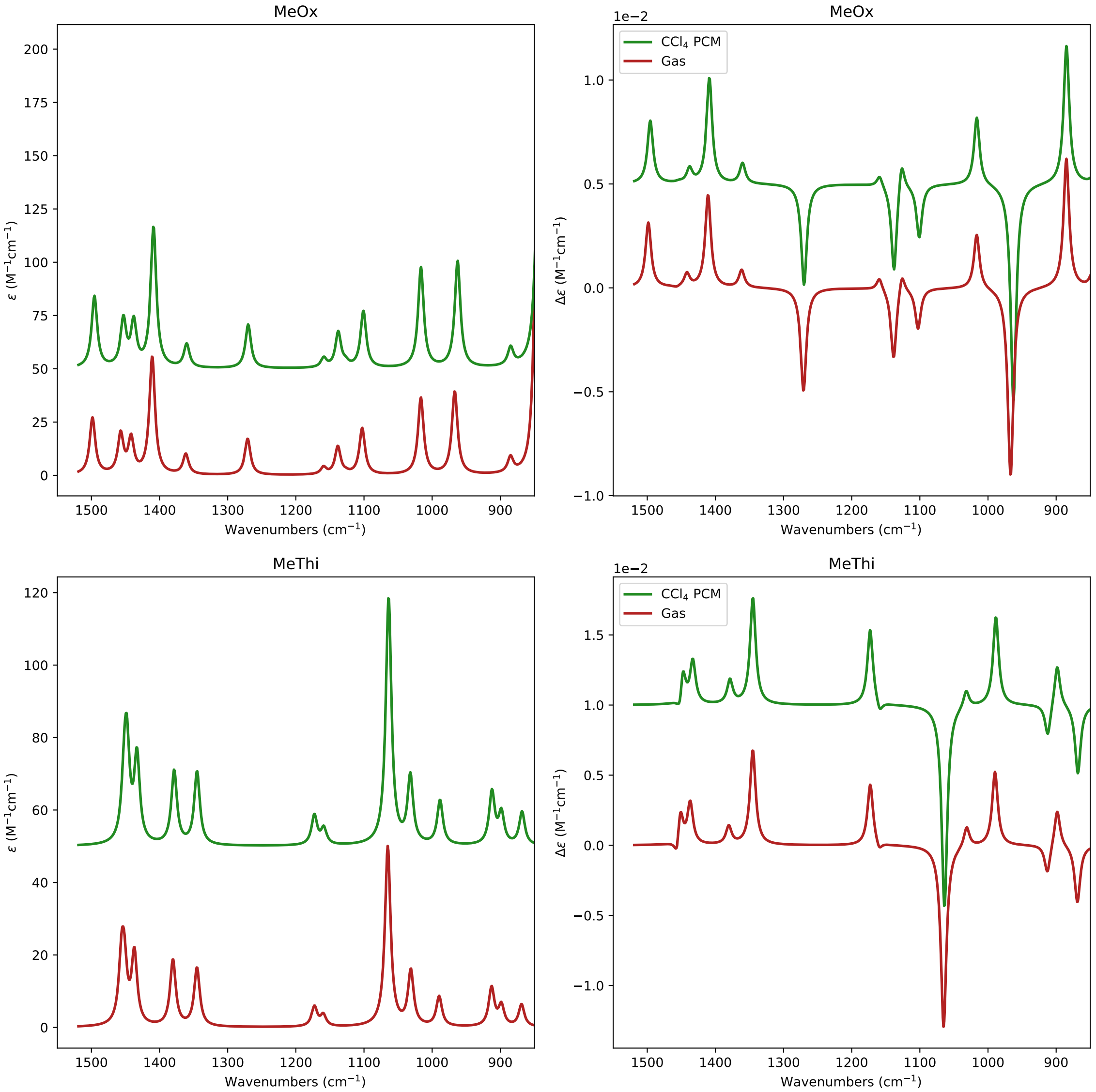

Figure S12: Comparison of harmonic IR and VCD spectra of (R)-2-methyloxirane (top panels) and (R)-2-methylthiirane (bottom panels) in the mid-IR region obtained with (green lines) and without (red lines) including CCl_4 solvent effects represented by the polarizable continuum model (PCM). Calculations were performed at the PW91 level of theory. The spectra were simulated by assigning Lorentzian distribution functions with 7 cm^{-1} of half-width at half-maximum. A scaling factor of 0.98 was applied.

Table S1: Assignment of normal modes of (*R*)-methyloxirane and (*R*)-methylthiirane, based on harmonic DFT calculations

	Methylthiirane		Methyloxirane	
Mode	ν (cm^{-1})	assignment	ν (cm^{-1})	assignment
1	230.94	CH_3 rotation	205.44	CH_3 rotation
2	300.79	CH_3 out of plane bending	366.51	CH_3 in plane bending
3	384.16	CH_3 in plane bending	409.31	CH_3 out of plane bending
4	620.72	C*S stretching	791.51	antisymmetric CO stretchings
5	654.80	CS stretching	864.40	CC stretching<br>+ CO stretching
6	886.52	CCH_3 stretching + CH_2 rocking	903.02	CH_2 rocking<br>+ C*H bending<br>CO stretching
7	916.76	CH_2 twisting	986.55	CO stretching<br>+ C*H bending<br>+CH_3 umbrella
8	931.41	CH_2 twisting<br>+ C*H bending<br>CS stretching	1037.27	CH_2 twisting<br>+ C*H bending<br>CO stretching
9	1009.91	CH_2 twisting<br>C*H bending<br>CS stretching	1125.13	CH_2 twisting<br>+ CCC bending<br>+ CH_3 antisymmetric bending
10	1052.31	CH_2 wagging	1149.24	CH_2 wagging
11	1086.83	CH_2 rocking + C*H bending<br>+ CS stretching	1161.60	CH_2 twisting<br>+ C*H out of plane bending
12	1183.42	CH_2 benging + CC stretching	1182.93	C*H out of plane bending
13	1196.64	C*H bending + CC stretching	1296.62	C*H in plane bending
14	1372.52	CH_2 bending + C*H bending<br>+ CH_3 antisymmetric bending<br>+ CC stretching	1389.24	CH_3 symmetric bending
15	1408.51	CH_3 umbrella<br>+ CCH_3 stretching<br>+ C*H bending	1439.55	CCH_3 stretching + C*H bending<br>+ CH_2 bending<br>+ CH_3 antisymmetric bending
16	1466.27	CH_3 antisymmetric bending<br>+ CH_2 scissoring	1471.16	CH_3 antisymmetric bending
17	1481.34	CH_2 scissoring<br>+ CH_3 antisymmetric bending	1486.80	CH_3 antisymmetric bending
18	1485.42	CH_3 antisymmetric bending	1528.99	CH_2 scissoring
19	3030.03	CH_3 symmetric stretching	3038.21	CH_3 symmetric stretching
20	3107.21	CH_3 antisymmetric stretching	3089.85	CH_2 symmetric stretching
21	3124.49	CH_2 symmetric stretching	3105.19	C*H stretching<br>+ CH_3 antisymmetric stretching
22	3129.57	CH_3 antisymmetric stretching<br>+ C*H stretching	3113.76	CH_3 antisymmetric stretching
23	3153.65	C*H stretching<br>+ CH_3 antisymmetric stretching	3133.88	CH_3 antisymmetric stretching<br>+ C*H stretching
24	3219.26	CH_2 antisymmetric stretching	3181.59	CH_2 antisymmetric stretching

S4 Anharmonic Simulations

Criteria used in identification of Fermi and Darling-Dennison resonances through two-step procedures:

- $1-2$ Fermi resonances: Maximum Frequency difference 200.0 cm^{-1}; Minimum Difference PT2 vs Variational 1.0 cm^{-1}; Intensity-specific term 1.0 cm (as defined in ref S3)

- $2-2$ Darling-Dennison resonances: Maximum Frequency difference 100.0 cm^{-1}; Minimum value of off-diagonal term 1.0 cm^{-1}

- $1-1$ Darling-Dennison resonances: Maximum Frequency difference 100.0 cm^{-1}; Minimum value of off-diagonal term 10.0 cm^{-1}; Intensity-specific term 1 cm

- $1-3$ Darling-Dennison resonances: Maximum Frequency difference 100.0 cm^{-1}; Minimum value of off-diagonal term 1 cm^{-1}; Intensity-specific term 0.025 cm

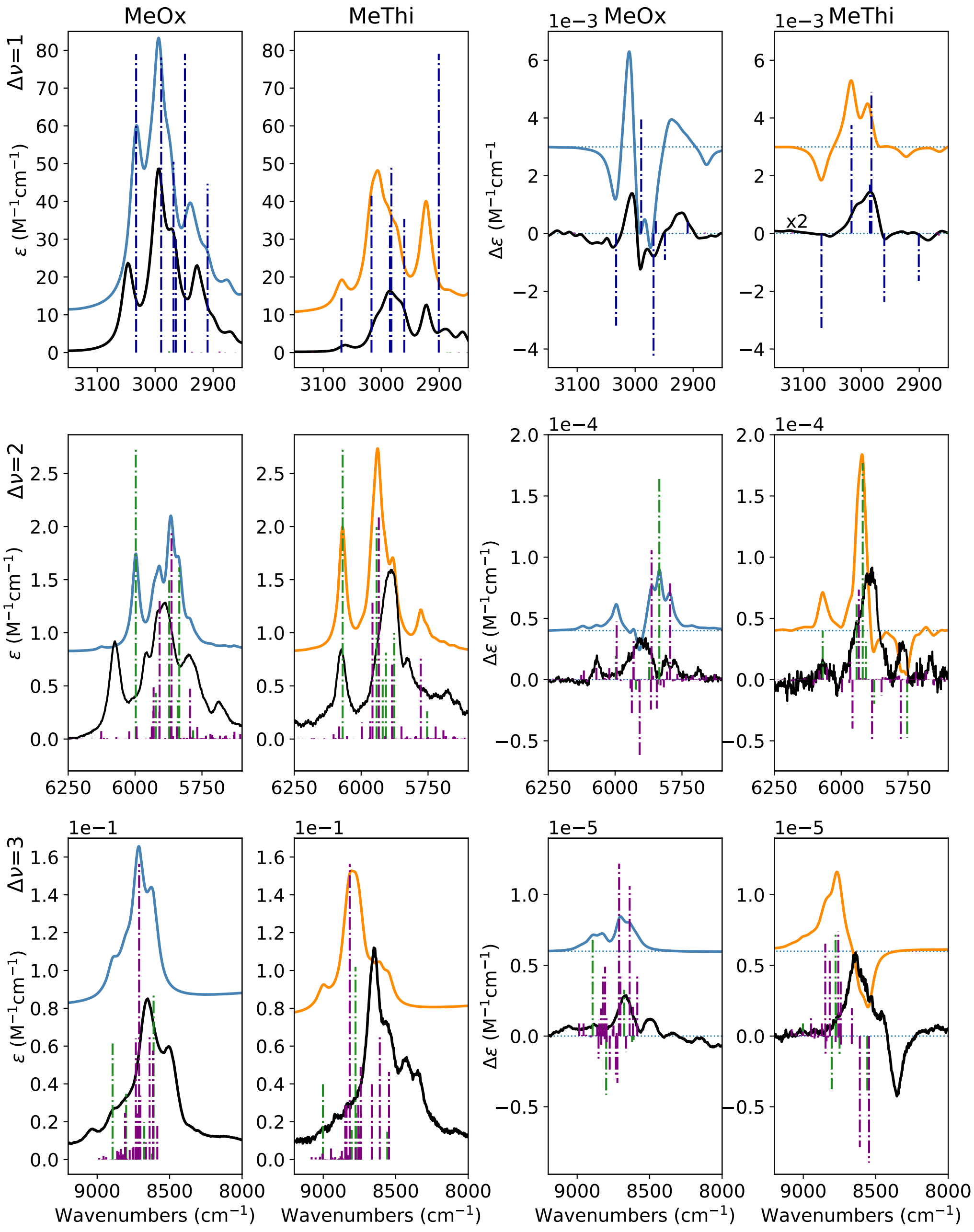

Figure S13: Comparison of the experimental (black lines) IR (first and second columns) and VCD (third and fourth columns) spectra of (R)-2-methylthiirane and (R)-2-methyloxirane with the corresponding anharmonic calculations in the CH regions. Calculations were performed at the PW91 level of theory. The spectra were simulated by assigning Lorentzian distribution functions with 10 cm^{-1}, 15 cm^{-1} and 40 cm^{-1} of half-width at half-maximum in the fundamental, first and second overtone regions, respectively. Different colors are used in the line spectra to separate transitions to fundamental (blue lines), overtones (green) and combination (purple) states.

S5 Local Mode Results

Following the procedure reported in refs. S4 and S5, ω_0 and χ were obtained performing an adequate number of z positive and negative displacements and evaluating quadratic ϕ_{ll} ($\partial^2 V/\partial z^2$), cubic ϕ_{lll} ($\partial^3 V/\partial z^3$), and quartic ϕ_{llll} ($\partial^4 V/\partial z^4$) force constants by a polynomial fitting to a Morse potential in the internal stretching coordinate z. For each CH bond, system is oriented in CH frame with the z-axis oriented along the CH bond and the y-axis lying on the plane defined by the C,C,H plane. 55 equally spaced mass-weighted steps were examined in the z-coordinate range [≈-0.33 Å, ≈0.5 Å] centered around equilibrium bond length and their energy evaluated through single-point calculation with Gaussian. The ϕ_{lll} and ϕ_{llll} were then evaluated by a polynomial fit with a 8th order polynomial.[S5] In order to decrease the computational time, Π and A were evaluated one every four points of the grid and fitted with a 8th order polynomial, whose coefficients were used in equations 3 and 4 of main text.

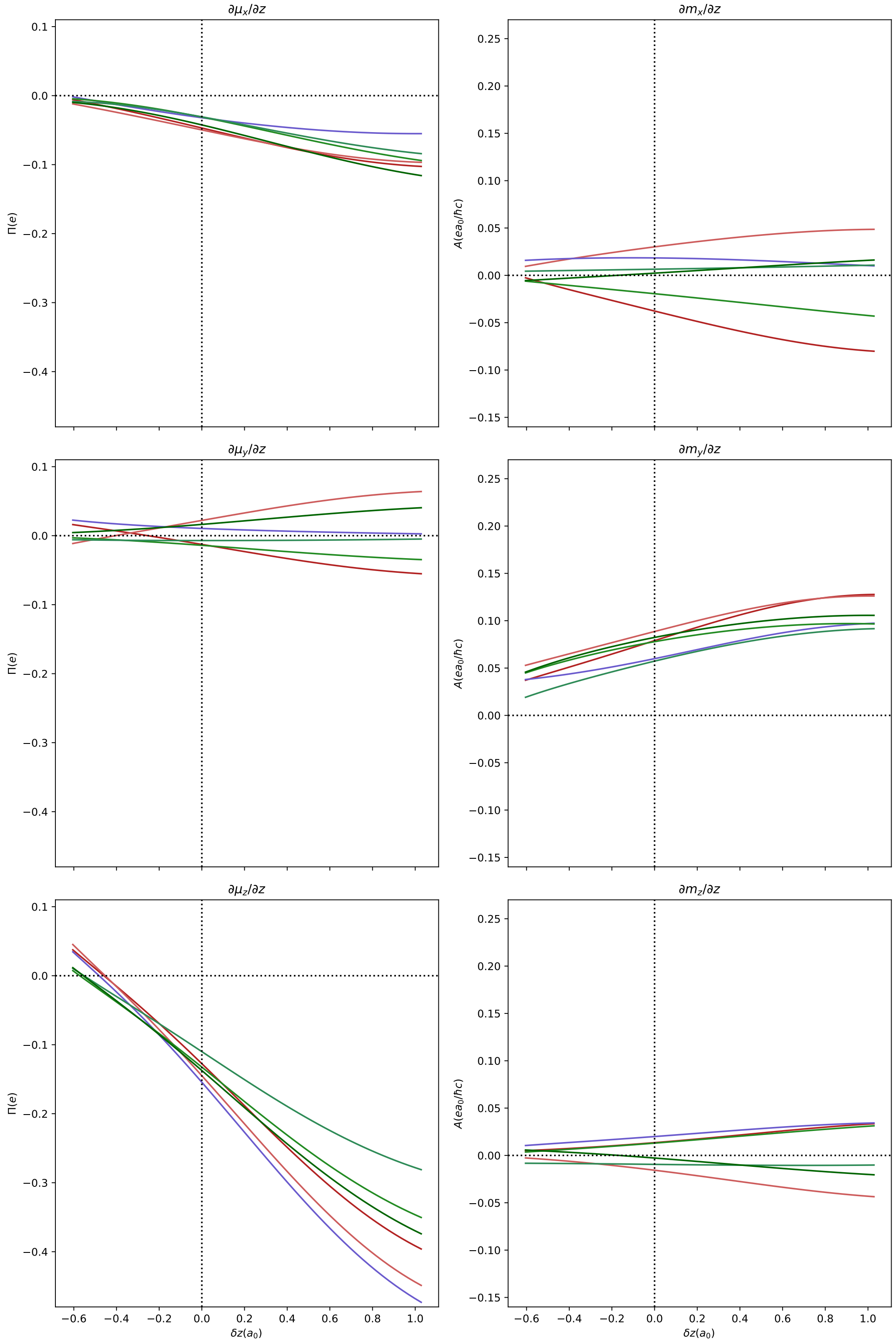

Figure S14: Dependence of the longitudinal and transverse components of the APTs and AATs for the Hydrogen atoms of (R)-2-methyloxirane with respect to the CH-bond length (z-direction) (see text). APTs are in atomic unit of charge e (electrons), AATs are in units of $(ea_0)/(\hbar c)$, where a_0 is the Bohr radius, and c the velocity of light. Displacements are in angstroms (Å). Green lines are used for CH_3 hydrogen atoms, red lines for CH_2 and the blue one for CH.

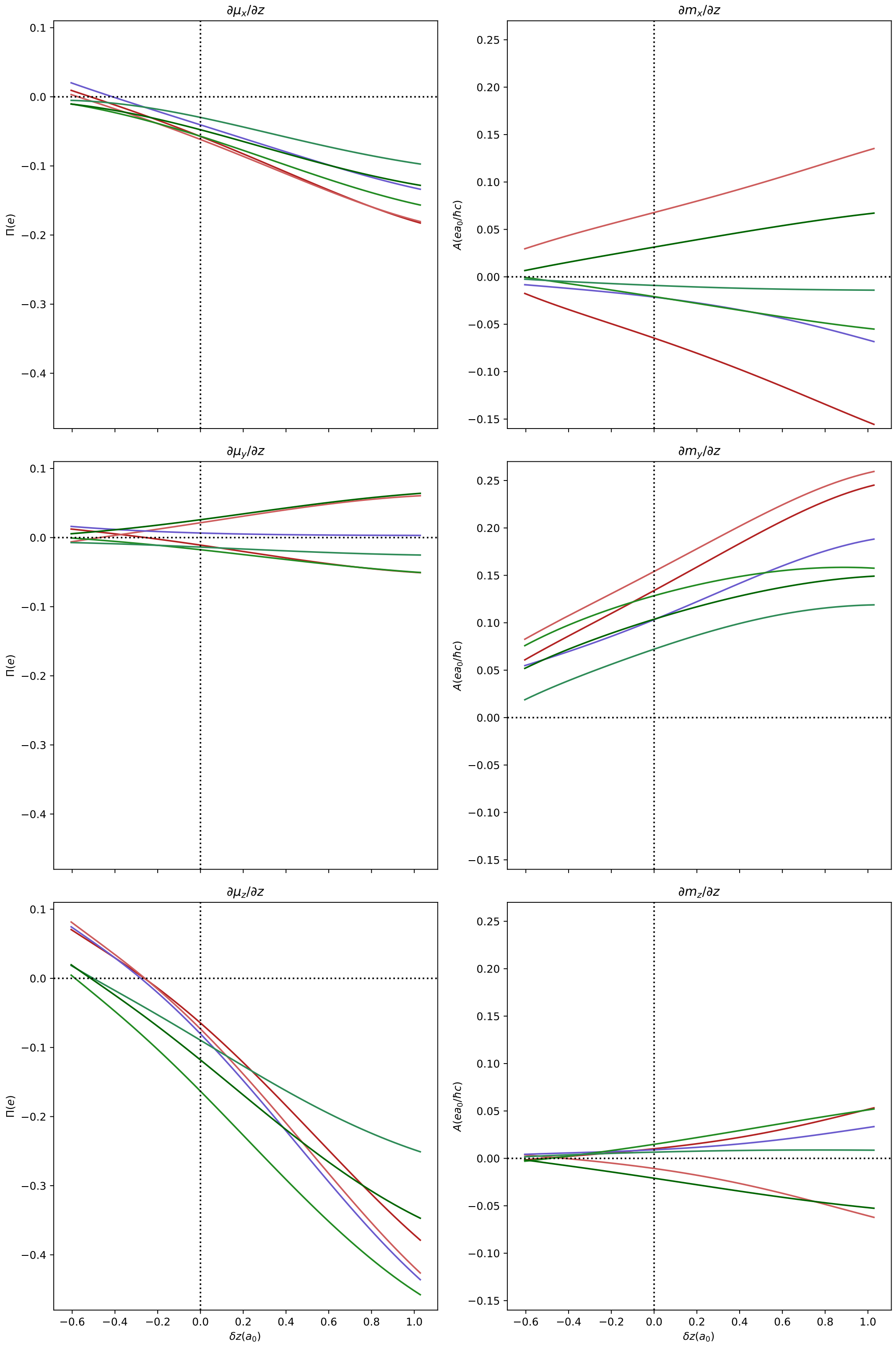

Figure S15: Dependences of longitudinal and transverse components of APTs and AATs of Hydrogen atoms for (R)-2-methylthiirane with respect to the CH-bond lenght (z-directions) (see text). APTs are in atomic unit of charge e (electrons), AATs are in units of $(ea_0)/(\hbar c)$, where a_0 is the Bohr radius, and c the velocity of light. Displacements are in angstroms (Å). Green lines are used for CH_3 hydrogen atoms, red lines for CH_2 and the blue one for CH.

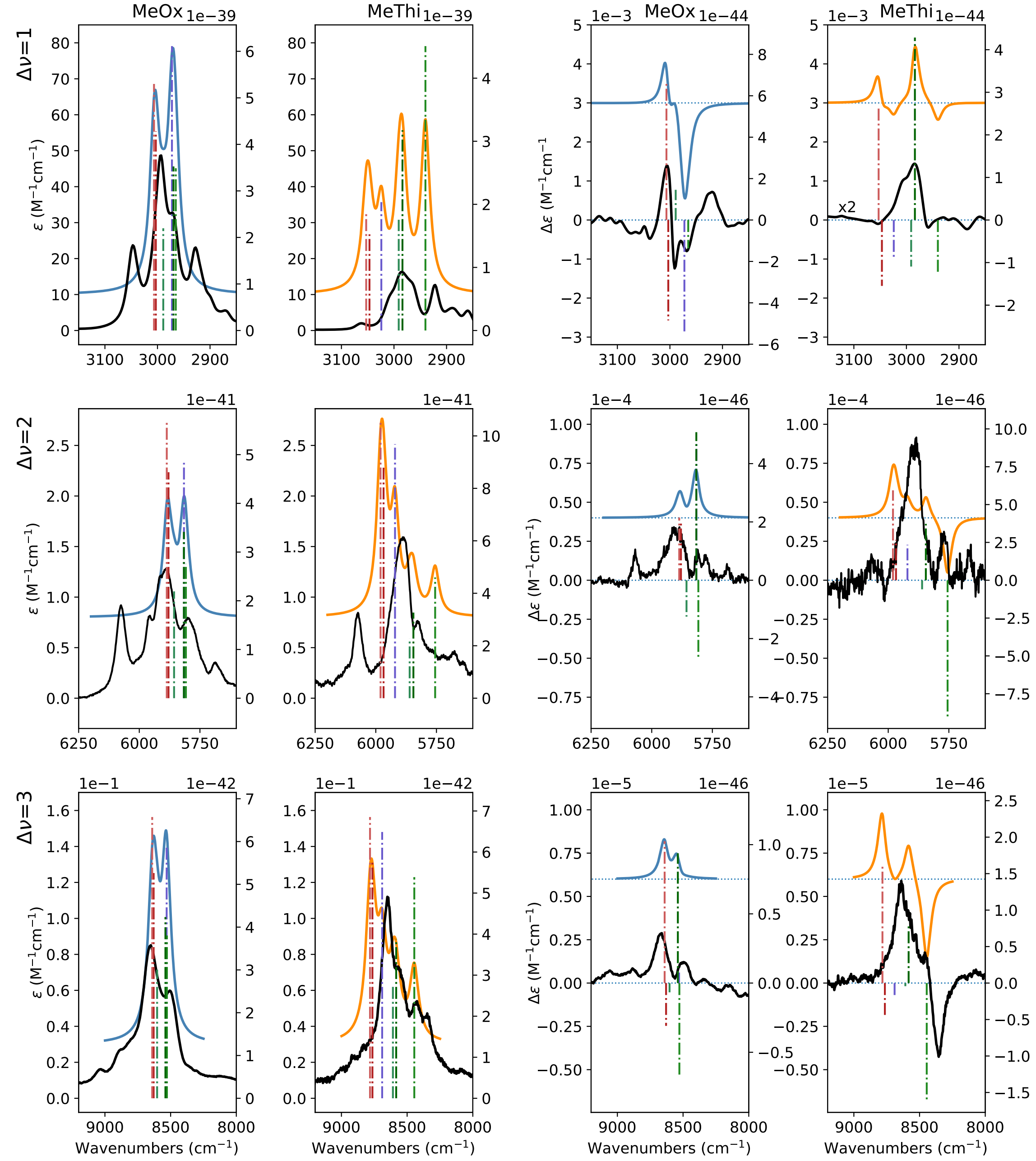

Figure S16: Comparison of the experimental IR (first and second columns) and VCD (third and fourth columns) spectra of (R)-2-methylthiirane (second and fourth columns, in orange) and (R)-2-methyloxirane (first and third columns, blue) with the corresponding local-mode calculations in the CH-stretching regions. The spectra were simulated by assigning Lorentzian distribution functions with 10 cm^{-1}, 15 cm^{-1} and 40 cm^{-1} of half-width at half-maximum in the fundamental, first and second overtone regions, respectively. The dipole and rotatory strengths are also reported, in cgs units. Green lines are used for CH_3 hydrogen atoms, red lines for CH_2 and blue for CH.

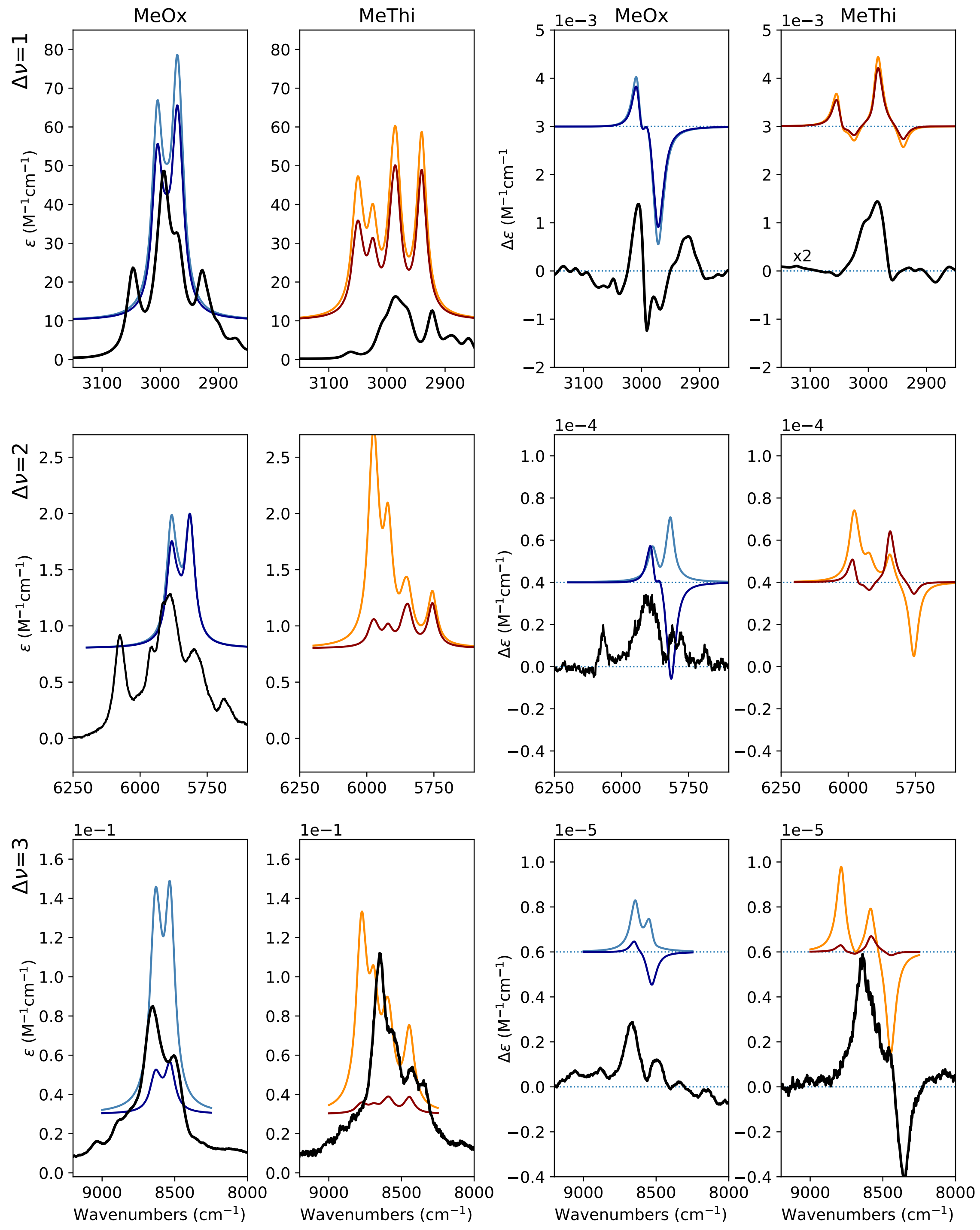

Figure S17: Comparison of the experimental IR (first and second columns) and VCD (third and fourth columns) spectra of (R)-2-methylthiirane (second and fourth columns, in orange) and (R)-2-methyloxirane (first and third columns, in blue) with the corresponding local-mode calculations in the CH-stretching regions. The spectra were simulated by assigning Lorentzian distribution functions with 10 cm^{-1}, 15 cm^{-1} and 40 cm^{-1} of half-width at half-maximum in the fundamental, first and second overtone regions, respectively. In red and deep blue lines are reported the spectra in wich only the zero-th order of equations 3 and 4 in the main text were considered, therefore removing the contribution of electrical anharmonicity.

S6 Cartesian Coordinates

Methylthiirane - B3PW91-D3BJ/SNSD

10

Enegy: -516.04096051

S	1.063457	-1.089446	-0.248587
C	0.935850	0.733200	-0.101037
H	0.781847	1.253161	-1.043715
H	1.662970	1.189320	0.565353
C	-0.217400	0.014668	0.483028
H	-0.231628	-0.048108	1.569986
C	-1.567200	0.070186	-0.177682
H	-2.108537	0.968192	0.147748
H	-1.463172	0.100138	-1.266431
H	-2.171395	-0.803498	0.084223

Methyloxirane - B3PW91-D3BJ/SNSD

10

Enegy: -193.06951416

O	0.811967	-0.729727	-0.140266
C	0.957848	0.689365	-0.115139
H	0.853937	1.180755	-1.082987
H	1.761684	1.061858	0.519586
C	-0.212523	0.045652	0.485531
H	-0.220773	-0.052539	1.573433
C	-1.557301	0.048003	-0.180515
H	-2.168748	0.876864	0.194304
H	-1.449647	0.153828	-1.264177
H	-2.091652	-0.886247	0.023115

References

(S1) Polavarapu, P. L.; Hess, B. A.; Schaad, L. J.; Henderson, D. O.; Fontana, L. P.; Smith, H. E.; Nafie, L. A.; Freedman, T. B.; Zuk, W. M. Vibrational spectra of methylthiirane. *J. Chem. Phys.* **1987**, *86*, 1140–1146.

(S2) Kleiner, C. M.; Horst, L.; Würtele, C.; Wende, R.; Schreiner, P. R. Isolation of the key intermediates in the catalyst-free conversion of oxiranes to thiiranes in water at ambient temperature. *Org. Biomol. Chem.* **2009**, *7*, 1397–1403.

(S3) Yang, Q.; Mendolicchio, M.; Barone, V.; Bloino, J. Accuracy and Reliability in the Simulation of Vibrational Spectra: A Comprehensive Benchmark of Energies and Intensities Issuing From Generalized Vibrational Perturbation Theory to Second Order (GVPT2). *Front. Astron. Space Sci.* **2021**, *8*, 665232.

(S4) Paoloni, L.; Mazzeo, G.; Longhi, G.; Abbate, S.; Fusè, M.; Bloino, J.; Barone, V. Toward Fully Unsupervised Anharmonic Computations Complementing Experiment for Robust and Reliable Assignment and Interpretation of IR and VCD Spectra from Mid-IR to NIR: The Case of 2,3-Butanediol and *trans* -1,2-Cyclohexanediol. *J. Phys. Chem. A* **2020**, *124*, 1011–1024.

(S5) Gangemi, F.; Gangemi, R.; Longhi, G.; Abbate, S. Calculations of overtone NIR and NIR-VCD spectra in the local mode approximation: Camphor and Camphorquinone. *Vib. Spectrosc.* **2009**, *50*, 257–267.
